# Harmful algal bloom species *Microcystis aeruginosa* releases thiamin antivitamins to suppress competitors

**DOI:** 10.1128/mbio.01608-25

**Published:** 2025-07-02

**Authors:** Mohammad Yazdani, Christopher P. Suffridge, Fangchen Liu, Cait M. Costello, Zhiyao Zhou, Gillian St. John, Ruchika Bhawal, Sheng Zhang, Geoffrey W. Coates, Mingming Wu, Beth A. Ahner

**Affiliations:** 1 Department of Biological and Environmental Engineering, Cornell University 251793, Ithaca, New York, USA; 2 Department of Microbiology, Oregon State University 549630, Corvallis, Oregon, USA; 3 Department of Chemistry and Chemical Biology, Cornell University 251791 , Ithaca, New York, USA; 4 Proteomics and Metabolomics Facility, Institute of Biotechnology, Cornell University 635264, Ithaca, New York, USA; University of Tennessee at Knoxville, Knoxville, Tennessee, USA

**Keywords:** bacimethrin, *Microcystis*, methoxythiamin, thiamin, antivitamins, exometabolome

## Abstract

In environmental ecosystems, vitamin concentrations are often exceedingly low, and auxotrophy, or reliance on exogenous vitamins or vitamin precursors, is widespread. We show here that the widespread harmful algal bloom (HAB) species *Microcystis aeruginosa,* threatening freshwater aquatic ecosystems globally, releases a complex mixture of thiamin antivitamins, including bacimethrin and methoxythiamin, which induce thiamin deficiency in the model green alga *Chlamydomonas reinhardtii*. Putative biosynthetic genes for bacimethrin were upregulated in *M. aeruginosa* when grown in co-culture, resulting in a greater production of bacimethrin. Bacimethrin, methoxythiamin, oxidized forms of thiamin and methoxythiamin, and a novel structural homolog of bacimethrin were all found at elevated levels in the co-culture exometabolome extracts and were all inhibitory to the growth of *C. reinhardtii* individually at very low concentrations and as a mixture in culture medium extracts. The thiamin-requiring mutant *C. reinhardtii*, CC-25, was much more sensitive to bacimethrin and methoxythiamin than the wild-type. Thiamin addition largely rescued the inhibitory effects of exposure to antivitamins in both the wild-type and mutant strains. Finally, we determined that bacimethrin is present in aquatic environments and is elevated during *Microcystis* blooms. Thus, allelopathic suppression of competitors, particularly those that are auxotrophic for thiamin, by *M. aeruginosa* via the production of antivitamins in environments where thiamin availability is low, could help this species to become dominant and form blooms.

**IMPORTANCE:**

The frequent reliance of aquatic microorganisms on exogenous vitamins leaves them potentially vulnerable to antimetabolites that mimic vitamins. We show that *Microcystis aeruginosa*, a common freshwater harmful algal bloom (HAB) species, makes and releases a chemical that mimics the required vitamin thiamin (vitamin B1) and one of its precursors. In the laboratory, these chemicals, along with related ones, can harm other algae. Production of these chemicals may help *Microcystis aeruginosa* thrive under conditions where thiamin is scarce and forms toxic blooms. HABs threaten and kill fish and other aquatic animals, as well as contaminate drinking water. Discovery of a role for antivitamins in freshwater HAB formation could lead to new strategies to prevent or control HABs.

## INTRODUCTION

Freshwater and marine ecosystems worldwide are increasingly threatened by harmful alga blooms (HABs), which can cause widespread fish, bird, and mammal death as well as major disruptions to drinking water supply and recreation (1, 2). HABs occur when toxin-producing species of cyanobacteria or microalgae out-compete non-toxic species for macronutrients, light, and other resources. Eutrophication is cited as a leading cause of HABs, and climate change appears to be increasing the scope and frequency of blooms; however, our understanding of factors leading to bloom formation and their persistence is far from complete (2–4).

Microbes in communities from diverse environments, including aquatic ecosystems where HABs occur, are known to exchange important cellular metabolites (5), including vitamins (6). Indeed, the exchange of vitamins is so prevalent that auxotrophy, or the complete reliance on an exogenous source of vitamin or vitamin precursors, is common in evolutionarily diverse species (7–9). In marine aquatic ecosystems, dissolved concentrations of required vitamins, such as thiamin, are also exceedingly low (10), and the bioavailability of vitamins has been hypothesized to influence plankton species diversity and succession in seawater (11–13). Less is known about the influence of vitamins on freshwater ecosystems, but the concentrations of thiamin in lakes and rivers may be similar to those in seawater (summarized in 14) or are in fact lower than in seawater (15, 16).

Also prevalent in microbial communities are allelopathic interactions, where one organism suppresses another by releasing an inhibitory chemical compound (17). Cyanobacterial species, such as *Microcystis aeruginosa,* which is responsible for toxic blooms in many freshwater systems, are known to release cyanotoxins (18) or lipophilic metabolites (19, 20) to inhibit non-toxic green algae that naturally compete for light and nutrients. However, evidence from field studies confirming that allelopathic interactions are instrumental in species succession or bloom formation is limited (20), and a role for allelopathy in dilute and turbulent marine ecosystems has been disputed (21). Due to the limited availability of vitamins in surface water and the strong likelihood of high-affinity vitamin transport proteins in many cell types (22, 23), allelopathic chemicals with structural similarity to vitamins or their precursors may be very effective inhibitors of algal and microbial activity.

Chemicals that are inhibitory or toxic because they mimic vitamins are called antivitamins. They can cause vitamin deficiency by interfering in biochemical pathways that require that particular vitamin. Most research on antivitamins has been focused on drug discovery (24), from which it is known that multiple bacterial species synthesize thiamin antivitamins (25–27). Biochemical synthesis of the antivitamin bacimethrin, which is an analog of the 4-amino-5-hydroxymethyl-2-methylpyrimidine (HMP) thiamin precursor, was first characterized in *Clostridium botulinum* (25). Upon uptake into cells, thiamin biosynthesis enzymes convert bacimethrin into 2′-methoxy-thiamin (or methoxythiamin) pyrophosphate, which inhibits thiamin-dependent enzymes required for growth (28).

Given that the highly successful freshwater cyanobacteria *M. aeruginosa* can synthesize its own thiamin, we hypothesize that an overlooked secret to its success might involve the production and release of thiamin antivitamins to which it is more resistant than its competitors. Here, we used a co-culture, including *M. aeruginosa* and the model green alga *Chlamydomonas reinhardtii,* to show that thiamin antivitamins are produced by *M. aeruginosa* and that the presence of the competing algae influences antivitamin accumulation. We also document elevated levels of one antivitamin during a *Microcystis* bloom and, using a *C. reinhardtii* mutant, confirm that species unable to make their own thiamin are likely to be particularly sensitive to this type of allelopathy.

## RESULTS

### Thiamin rescues growth inhibition of *C. reinhardtii* by *M. aeruginosa*

**Fig 1:**
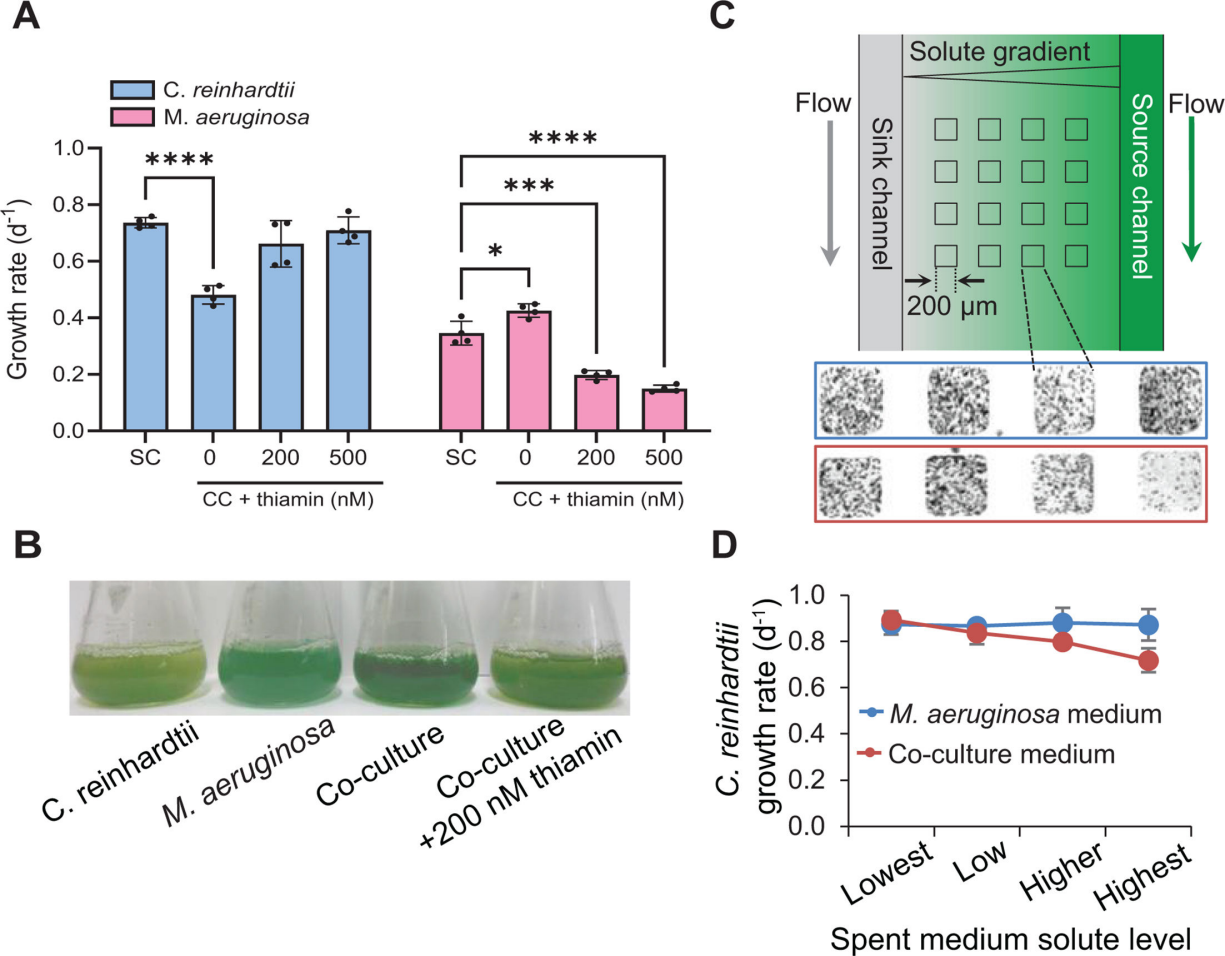
(**A**) Growth rate of *C. reinhardtii* and *M. aeruginosa* in single culture (SC) and co-culture (CC) with and without thiamin. (**B**) At t = 10 d, from left to right, single cultures of *C. reinhardtii* and *M. aeruginosa*, and the co-cultures without (blue-green) and with thiamin (yellow-green). (**C**) Schematic of the microfluidic device used to generate spent-medium exudate gradient in replicate microhabitats. Epi-fluorescent microscope images of cells in microhabitats exposed to *M. aeruginosa* spent medium (blue box) and co-culture medium (red box) at t = 3 d. (**D**) *C. reinhardtii* growth rate in individual microhabitats. For A and D, data are means ± SEM, with *n* ≥ 3. Asterisks indicate significance in two-way ANOVA with Tukey post-hoc test (*P < 0.05, ***P < 0.0002, ****P < 0.0001).

Cyanobacteria produce many antimetabolites, some of which are toxic to photosynthetic eukaryotes (18–20, 29). Accordingly, when we cultured *M. aeruginosa* together with *C. reinhardtii* in minimal medium BG-11, it was not surprising that the latter grew 35% slower than when grown alone (Fig. 1A). In contrast, *M. aeruginosa* grew at a rate that was 23% higher than when grown alone (Fig. 1A) and ultimately dominated the co-culture after 10 days (Fig. 1B). To determine whether exudates were responsible for the observed inhibition, we evaluated the growth of *C. reinhardtii* in a microfluidic device that can be used to expose cells to a steady-state gradient of solutes (30) (Fig. 1C). *C. reinhardtii* cells were seeded in an array of microhabitats patterned into an agarose membrane, and a chemical gradient was established via diffusion by pumping spent and fresh medium into the source and sink channels, respectively. After 3 days, microscopy showed significantly fewer *C. reinhardtii* cells in the microhabitats exposed to higher solute levels from the co-culture spent medium (rightmost microhabitat in red box, Fig. 1C) than those closer to the sink channel. The growth rate was also the lowest in these microhabitats (Fig. 1D). In contrast, the growth rate of *C. reinhardtii* was the same in all the microhabitats exposed to a gradient of solutes from the spent medium of *M. aeruginosa* culture (Fig. 1D).

Given that one previously characterized allelopathic interaction between *M. aeruginosa* and alga-induced oxidative stress (20), we added thiamin, a vitamin known to enhance plant tolerance to oxidative stress (31), to a subsequent set of co-cultures. In the presence of thiamin, the growth rate of *C. reinhardtii* fully recovered, whereas the *M. aeruginosa* growth rate decreased (Fig. 1A), resulting in a co-culture dominated by *C. reinhardtii* instead of *M. aeruginosa* (Fig. 1B). Thiamin addition to single cultures of *M. aeruginosa* did not cause inhibition, whereas *C. reinhardtii* growth was slightly enhanced (Fig. S1). Given the strong link we observed between thiamin addition and co-culture domination, we decided to test an alternate hypothesis regarding thiamin’s role in mitigating allelopathy: the production of thiamin antivitamins by *M. aeruginosa*.

### *M. aeruginosa* produces thiamin antivitamins

**Fig 2:**
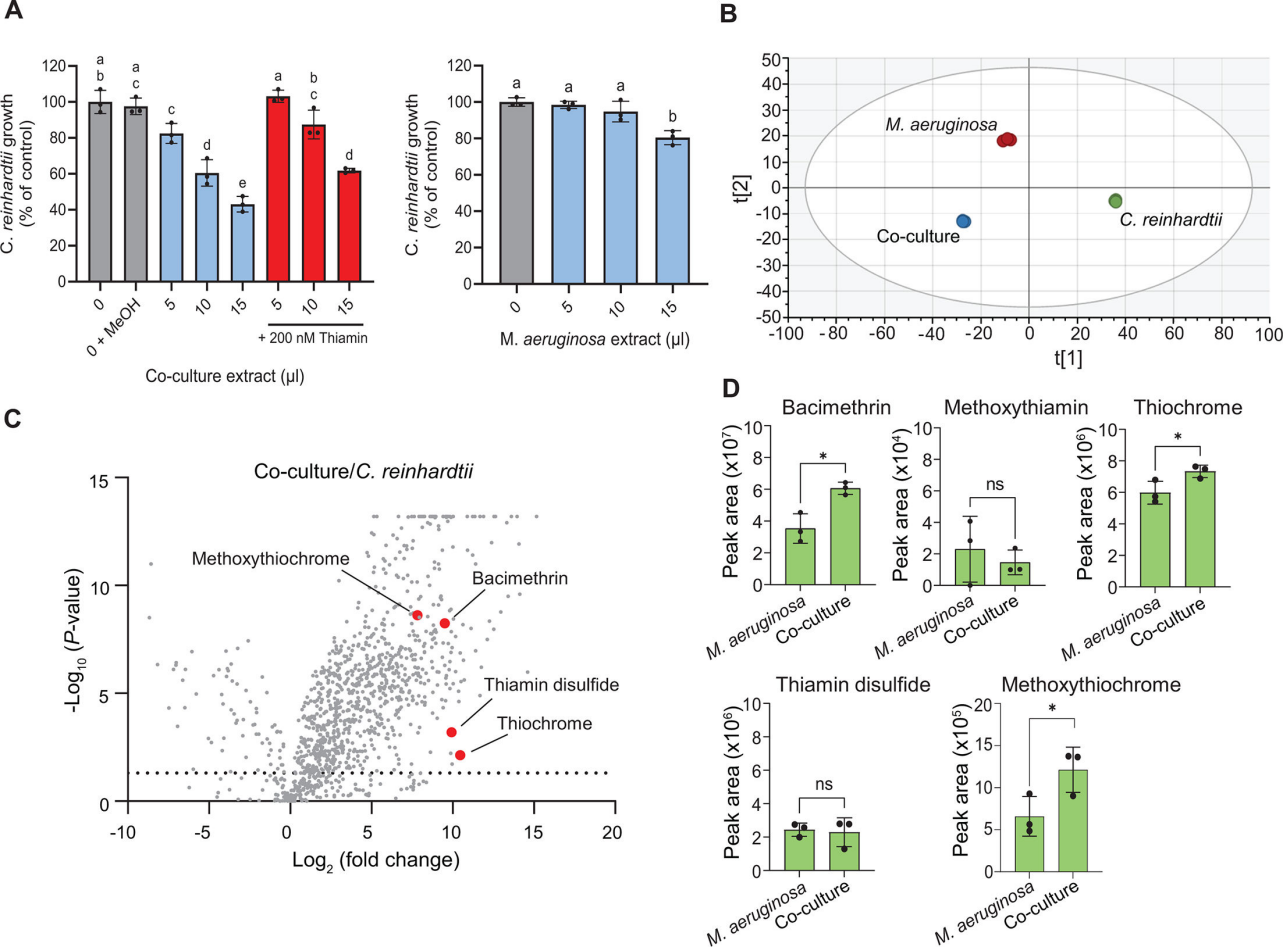
(**A**) Relative growth rate of *C. reinhardtii* with added co-culture exometabolomic extract without and with added thiamin (left) and with *M. aeruginosa* culture extract (right). Controls are with no addition or 15 µL methanol (+MeOH). (**B**) Scatter plot showing Orthogonal Projections to Latent Structures Discriminant Analysis (OPLS-DA) of peak areas for annotated metabolites detected via untargeted LC-MS in triplicate methanol extracts. (**C**) Volcano plot of metabolites in co-culture extracts relative to *C. reinhardtii*. Dots are calculated from triplicate measurements. The dotted line is *P* = 0.05. Thiamin antivitamins are shown as red dots. (**D**) Levels of thiamin antivitamins in *M. aeruginosa* single culture and co-culture extracts were measured by targeted LC-MS/MS using parallel reaction monitoring (PRM). For A and D, data are means ± SEM, with *n* = 3. Letters indicate significance in one-way ANOVA (*P* < 0.0001) with Tukey post-hoc test. Asterisks indicate significance in student’s *t*-test (*P < 0.05; ns: not significant).

Spent medium from the co-culture and single cultures of both species was lyophilized, and the chemical residuals were resuspended in methanol to further study the composition of the *M. aeruginosa* exudates. The co-culture methanol extract, like the spent medium in the microfluidic device, suppressed *C. reinhardtii* growth by up to 50% of controls, whereas *M. aeruginosa* single culture extract only reduced its rate by 20% (Fig. 2A). In contrast, the co-culture extract did not affect *M. aeruginosa* growth (Fig. S2). When thiamin was added with the co-culture extract, significantly less inhibition of *C. reinhardtii* was observed (Fig. 2A). Non-targeted liquid chromatography mass spectrometry (LC-MS/MS) analysis of methanol extracts was used to characterize the set of metabolites shed by cells into the growth medium, that is, the exometabolomes (Dataset S1). An orthogonal projection to latent structures discriminant analysis (OPLS-DA) plot of the 1,145 annotated compounds in the exometabolomes revealed that the co-culture exometabolome overlaps with the two derived from the individual cultures but is not simply a composite of the two (Fig. 2B).

Volcano plots comparing the individual exometabolomes reveal that 891 and 86 compounds, common to both the co-culture to the single *C. reinhardtii* culture extracts, were present in significantly higher and lower amounts, respectively (Fig. 2C). A similar pattern was observed (839 metabolites in greater abundance versus 56 lower) when comparing the exometabolomes of solo-cultured *M. aeruginosa* and *C. reinhardtii* (Fig. S3A). Many of the annotated chemicals are expected *M. aeruginosa* metabolites or metabolites observed in other microbial exometabolomes (32, 33). No thiamin was observed in any of the exometabolomes.

Bacimethrin, a thiamin antivitamin precursor, was observed in all the original exometabolomes, but the levels were 730-fold and 640-fold higher in the co-culture and single-culture *M. aeruginosa* extracts than in the extracts of the *C. reinhardtii* medium (red dots in Fig. 2C; Fig. S3A). The chemical identity of bacimethrin was confirmed by comparing its MS/MS fragmentation pattern with that of a standard (Fig. S4 and S5B). Levels of bacimethrin measured during targeted LC-MS/MS were roughly 70% higher in co-culture extracts than in single-culture extracts (Fig. 2D). Subsequent *C. reinhardtii* extracts, prepared in baked-clean glassware, contained no measurable bacimethrin (Fig. S5A).

**Fig 3:**
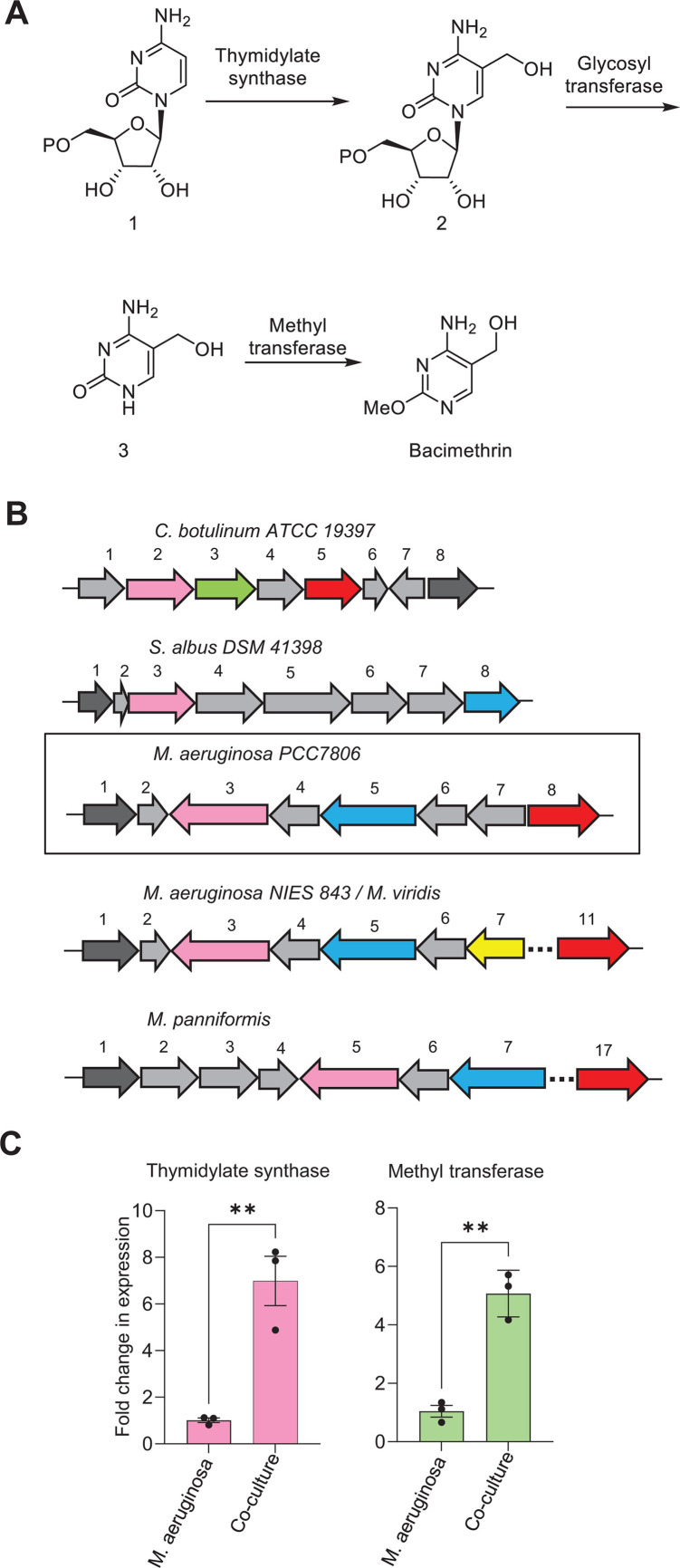
(**A**) Bacimethrin biosynthesis pathway in *C. botulinum* (*CB*) adapted from reference 25. Substrate 1: cytidine 5′-monophosphate, 2: 5-hydroxymethylcytidine 5′-monophosphate, 3: 5-hydroxymethylcytosine. (**B**) Gene cluster for bacimethrin biosynthesis in *CB* strain A (ATCC 19397), *Streptomyces albus* DSM 41398 (*SA*), and putative bacimethrin biosynthesis gene clusters in *M. aeruginosa (MA*) and two other *Microcystis* species (https://www.genome.jp/kegg/). In *CB*, 1- hypothetical protein (HP), 2- thymidylate synthase (TS), 3- methyltransferase (MT), 4- thiamin pyridinylase I, 5- phosphomethylpyrimidine kinase (ThiD2), 6- HP, 7- putative transporter, 8- ABC transporter (ABC). In *SA*, 1-ABC, 2- HP, 3- TS, 4- nucleoside phosphate kinase, 5- radical SAM domain protein, 6- DNA polymerase, 7- HP, 8- oxidoreductase. In *MA PCC7806*, 1- ABC, 2- HP, 3- TS, 4- thymidylate kinase (TK), 5- Mercuric reductase (MR), 6- HisIE, 7- uncharacterized gene, 8- ThiD2. In *MA NIES 843* and *M. viridis*, 1- ABC, 2- HP, 3- TS, 4- TK, 5- MR, 6- HisIE, 7- glycosyl transferase, 11- ThiD2. In *M. panniformis*, 1- ABC, 2- O antigen permease protein, 3- O antigen transport protein, 4- HP, 5- TS, 6- TK, 7- MR, 17- ThiD2. (**C**) Relative expression of putative bacimethrin biosynthetic genes in *MA* PCC7806 determined by RT-qPCR analysis. NCBI protein ID: thymidylate synthase (WP_002743898.1), and methyltransferase (WP_002744668). For C, data are means ± SEM, with *n* = 3. Asterisks indicate significance in a student’s *t*-test (**P < 0.01).

Bacimethrin is known to be toxic to many bacteria and yeast (25–27). Its biosynthetic pathway was elucidated in *C. botulinum* (25, reproduced in Fig. 3A), and all three of the required genes were reported to be co-located in one cluster. An altered version of this gene cluster for the same strain is now available in KEGG (34) where only two of the three genes (thymidylate synthase shown in pink and methyl transferase shown in green; Fig. 3B) are located in a gene cluster. In *Streptomyces albus*, a bacteria known to make bacimethrin (26), thymidylate synthase is not co-located with methyl transferase or glycosyl transferase (Fig. 3B), but in other bacteria, all three genes are also reported to be in the same cluster (35). Thymidylate synthase in *Microcystis* species has low homology with a bacterial version of the gene (≤30% as determined by blast analysis of the published protein sequences) and appears 50% larger than versions found in bacteria (Fig. 3B). Similar to bacimethrin gene clusters in *C. botulinum* and *S. albus*, *Microcystis* clusters also all contain an ABC-transporter (shown in black), and similar to *C. botulinum*, also all contain a copy of ThiD2 (shown in red), a version of ThiD that catalyzes the phosphorylation of HMP monophosphate, but not HMP or its toxic analogs (36). In two *Microcystis* species (*M. aeruginosa* NIES −843 and *M. viridis*), a glycosyl transferase (shown in yellow) is present in the gene cluster; in PCC7806, the *M. aeruginosa* strain used in this study, a pseudogene is found in place of the glycosyltransferase (gene 7, Fig. 3B). *M. panniformis* contains a gene cluster that is similar to the other *Microcystis* species but has some gene additions and deletions. In all four *Microcystis* gene clusters, there is an oxidoreductase, which, in some genomes, is annotated as a mercuric reductase (shown in blue, Fig. 3B).

Using RT-qPCR, we found that the thymidylate synthase and an upstream methyltransferase in *M. aeruginosa* PCC7806 were upregulated by 7-fold and 5-fold, respectively, when grown in co-culture compared with single culture (Fig. 3C). The observed gene upregulation correlated with higher levels of bacimethrin accumulation in the co-culture (Fig. 2D), which supports an allelopathic role for bacimethrin production. As noted for thymidylate synthase, there was little homology between the methyl transferase and glycosyl transferase genes in *Microcystis* and those found in bacteria. Because there were multiple glycosyl transferases in the PCC7806 genome and a pseudogene in place of the glycosyl transferase found in the other *M. aeruginosa* strain’s gene cluster, we were not able to quantify its expression in these experiments.

**Fig 4:**
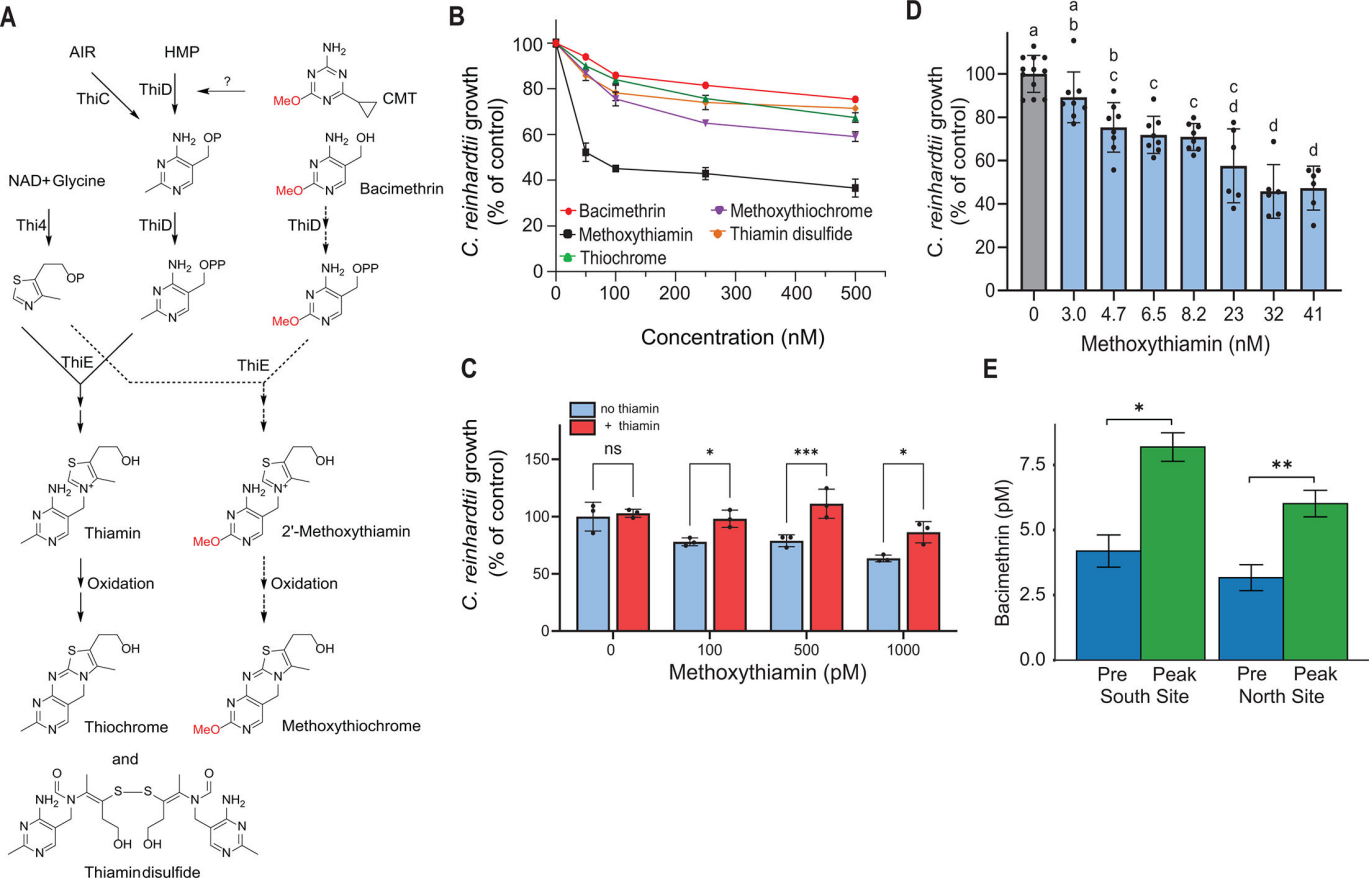
(**A**) Partial thiamin biosynthesis pathway for algae shown with known antivitamin integration steps (28), proposed inhibition by CMT, and structures of confirmed oxidation products. Enzyme and metabolite abbreviations: AIR, 5-aminoimidazole ribotide; HMP, 4-amino-5-hydroxymethyl-2-methylpyrimidine; ThiC, phosphomethylpyrimidine synthase; ThiD, HMP (phosphate) kinase; Thi4, thiazole synthase; and ThiE, thiamin-phosphate pyrophosphorylase. (**B**) Average relative growth rates (*n* = 3) of *C. reinhardtii* with thiamin antivitamins added separately, shown with thiamin additions in Fig. S9. (**C**) Average relative growth rates (*n* = 3) of *C. reinhardtii* in the presence of low methoxythiamin concentrations with no thiamin or with thiamin added at concentrations 10-fold lower than methoxythiamin (10, 50, and 100 pM, respectively). Thiamin added to the control (no methoxythiamin) was 10 pM. (**D**) Average relative growth rates of wild-type *C. reinhardtii* exposed to methoxythiamin in microfluidic device. In (**C**), asterisks indicate a statistically significant difference between thiamin and no thiamin using two-way ANOVA with Tukey post-hoc test (*P < 0.05, ***P < 0.001). In (**D**), letters indicate significance in one-way ANOVA (*P* < 0.0001) with the Tukey post-hoc test. (**E**) Dissolved bacimethrin concentrations in Upper Klamath Lake, Oregon. Bacimethrin was measured at the south and north ends of the lake (42.31046 N, 121.84369 W; and 42.46121 N 121.96013 W, respectively) in pre-bloom (blue bars, May 2023) and peak-bloom (green bars, August 2023) periods. Asterisks indicate statistically significant differences between sampling periods based on a paired *t*-test (*P = 0.011, **P = 0.0206). Bars are means (*n* = 3) ±StDEV.

Methoxythiamin is formed when bacimethrin is entrained into the thiamin biosynthesis pathway via ThiD (28) (Fig. 4A), but it was not observed initially in our exometabolomes. However, using targeted LC-MS/MS and a synthesized standard (prepared following the method described in reference 37, with modifications) (Fig. S6A), the presence of methoxythiamin was confirmed in the extracts derived from co-culture and *M. aeruginosa* single culture (Fig. S4 and S7). No difference between the levels of methoxythiamin in the single culture and co-culture extracts was observed, and extracted ion chromatogram (XIC) peak areas were roughly three orders of magnitude lower than those of bacimethrin (Fig. 2D). These results suggest that methoxythiamin is not the primary allelopathic chemical in our extracts, although it is possible that methoxythiamin is transient in the culture medium, perhaps because of chemical instability and/or rapid uptake by *C. reinhardtii* in the co-culture.

Because thiamin can be transformed into the oxidation products thiochrome and thiamin disulfide via enzymatic or chemical reactions (38, 39), we hypothesized that methoxythiamin could likewise be oxidized to methoxythiochrome or methoxythiamin disulfide (Fig. 4A). Indeed, we found measurable levels of methoxythiochrome, as well as thiochrome and thiamin disulfide, in our exometabolomes at significantly higher concentrations in the co-culture and *M. aeruginosa* extracts compared with those of *C. reinhardtii* (Fig. 2C; Fig. S3A). Compound identity was confirmed using standards of thiochrome and thiamin disulfide (Fig. S4, MS/MS of thiochrome only in Fig. S8A), and we synthesized methoxythiochrome (Fig. S6B) to confirm its presence with targeted LC-MS/MS (Fig. S4 and S8B). Interestingly, the methoxythiochrome XIC peak areas were 10-fold higher than methoxythiamin but still 100-fold lower than those of bacimethrin, and like bacimethrin, the levels were significantly higher in the co-culture extract (Fig. 2D). Thiochrome and thiamin disulfide peak areas were both roughly 10-fold lower than bacimethrin, but only thiochrome was higher in the co-culture extracts relative to *M. aeruginosa* (Fig. 2D). We found no evidence for methoxythiamin disulfide in any extracts.

### Thiamin antivitamins and oxidation products inhibit *C. reinhardtii,* but not *M. aeruginosa*

We used batch cultures to confirm that the thiamin antivitamins were toxic to *C. reinhardtii* and also to show that the various oxidation products were inhibitory. The chemicals were added individually to the growth medium with and without supplemental thiamin. Methoxythiamin was the most toxic to *C. reinhardtii*; at the highest concentrations, the growth of *C. reinhardtii* was reduced by roughly 60% (Fig. 4B), and we saw significant inhibition as low as 1 nM (Fig. 4C). In comparison, methoxythiochrome was less inhibitory, only reducing growth by 40% at the highest concentration. Bacimethrin, thiochrome, and thiamin disulfide were all similarly inhibitory, causing modest reductions in growth at the highest levels (~15%–25%, Fig. 4B). Adding excess thiamin (200 nM) reversed the inhibitory effect of all the thiamin-sized antivitamins except when the concentration of the antivitamin exceeded that of thiamin (Fig. S9A), and very low concentrations of thiamin (10-fold lower than antivitamin, 10-100 pM) reversed inhibition at low methoxythiamin concentrations (Fig. 4C). In contrast, thiamin addition fully reversed the inhibitory effects of bacimethrin, even when bacimethrin was added at 60-fold higher concentration (Fig. S9A). It is likely that bacimethrin uptake by *C. reinhardtii* is facilitated by hydroxymethyl pyrimidine (HMP) transporters, which may be downregulated in the presence of thiamin, whereas the methoxythiamin and oxidation products are in competition with the added thiamin for uptake by thiamin transporters. Of these five chemicals, only the methoxythiamin was somewhat inhibitory to *M. aeruginosa* (~25% growth reduction at 250 nM), and thiamin did not reverse inhibition (Fig. S9B). The primary source of the oxidized thiamin compounds is presumably *M. aeruginosa* since levels are similar in the co-culture and single-culture extracts (Fig. 2D).

**Fig 5:**
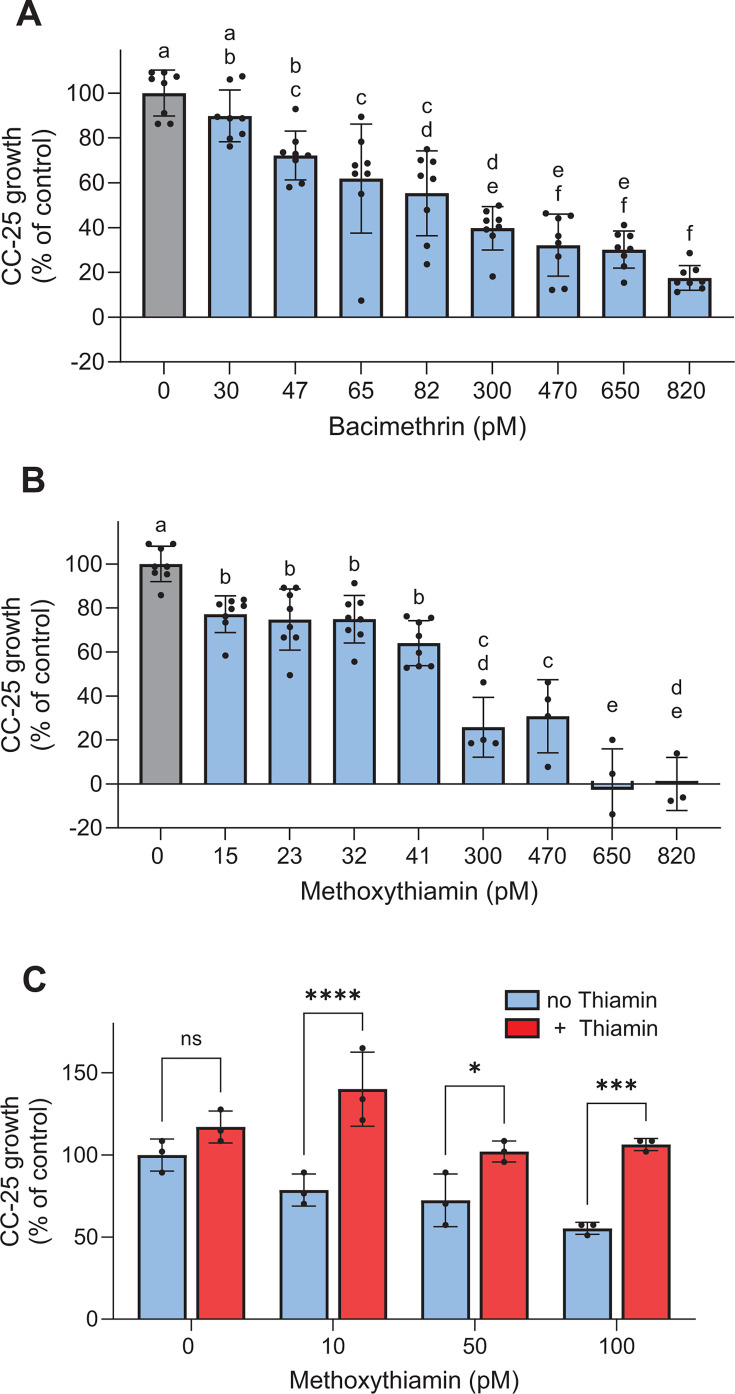
(**A**) Average relative growth rates of CC-25 exposed to bacimethrin in the microfluidic device. (**B**) Average relative growth rates of the same exposed to methoxythiamin. (**C**) Average relative growth rates (*n* = 3) of CC-25 in the presence of low methoxythiamin concentrations in batch culture with no thiamin or with thiamin added at concentrations 10-fold lower than methoxythiamin (1, 5, and 10 pM, respectively). Thiamin added to the control (no methoxythiamin) was 10 pM. In (**A** and **B**), letters indicate significance in one-way ANOVA (*P* < 0.0001) with Tukey post-hoc test, and in (**C**), asterisks indicate statistically significant differences in a two-way ANOVA between thiamin and no thiamin-treated samples (*P < 0.05, ***P < 0.0002, ****P < 0.0001).

We also grew *C. reinhardtii* in the microfluidic device in order to maintain very low steady-state concentrations of the antivitamins. We observed statistically significant reductions in growth at methoxythiamin concentrations as low as 4.7 nM. The growth reduction was positively correlated to methoxythiamin concentration, ultimately leveling out at about 50% of control (Fig. 4C), similar to the batch culture experiments (Fig. 4B). We repeated the batch and microfluidic device experiments with the *C. reinhardtii* thi8 mutant CC-25 (40), which lacks the ability to synthesize the pyrimidine moiety of thiamin. CC-25 cells were much more sensitive to both bacimethrin and methoxythiamin than the wild-type. We observed a significant reduction in growth rate at 47 pM bacimethrin (compared with approximately 100 nM in the wildtype) and nearly complete inhibition as the concentration neared 1 nM (Fig. 5A). In response to methoxythiamin, we saw a significant decrease in growth rate at concentrations as low as 15 pM, with complete cessation of growth occurring at 650 pM and higher (Fig. 5B). As with the wild-type cells, very low concentrations of thiamin completely reversed inhibition in batch experiments (1–10 pM; Fig. 5C).

### A novel antivitamin also inhibits *C. reinhardtii*

Among the many chemicals elevated in the exometabolomes of *M. aeruginosa*, we confirmed the identity of one as 4-cyclopropyl-6-methoxy-1,3,5-triazin-2-amine (CMT) (Fig. S4, S10A through C). CMT is homologous to HMP and bacimethrin and was particularly abundant in both co-culture and *M. aeruginosa* exometabolomes (Fig. S3B). Both the untargeted and targeted MS revealed significantly more CMT in the co-culture extracts than in the *M. aeruginosa* extracts (Fig. S11A). CMT mirrored bacimethrin in its inhibition of *C. reinhardtii,* and likewise, thiamin addition eliminated its inhibitory effects (Fig. S11B). This suggests that like bacimethrin, CMT is taken up via the HMP pathway and is perhaps inhibitory to ThiD, the enzyme that converts HMP to HMP-P (Fig. 4A) or other enzymes inhibited by bacimethrin (28). However, CMT is also structurally similar to the nonspecific triazine herbicide prometon, which blocks electron transport in photosystem II (41). We could find no reference to the biological production of CMT, although biosynthesis of cyclopropyl moieties is known (42), and we note that several other putative cyclopropyl-containing chemicals were annotated in the *M. aeruginosa* exometabolomes, including the known phytotoxin hypoglycin A, found in high levels in unripened lychee fruit (43). CMT was below detection in the *C. reinhardtii* extracts (Fig. S10A), and CMT did not inhibit *M. aeruginosa* (Fig. S11B).

### Bacimethrin is present in aquatic environments impacted by *Microcystis* blooms

As an environmental validation of our laboratory investigation, we determined that dissolved bacimethrin was present in Upper Klamath Lake, Oregon, a hypereutrophic system that experiences annual cyanobacterial HABs (44–46). During the peak algal bloom period, observed bacimethrin concentrations were 8.2 ± 0.5 and 6.0 ± 0.5 pM at the south and north ends of the lake, respectively, roughly double those observed during the pre-bloom period (Fig. 4E). Bacimethrin has not previously been reported in the environment. These data provide evidence that at least one of the antivitamins described herein is present in the environment and further support our hypothesis that *M. aeruginosa* produces antivitamins to gain a competitive advantage.

## DISCUSSION

We have documented here using co-culture cultivation and targeted MS that the HAB-forming freshwater cyanobacteria *M. aeruginosa* synthesizes the thiamin antivitamins bacimethrin and methoxythiamin. Toxic to a wide range of organisms (47), these chemicals are likely used by *M. aeruginosa* to compete with other photosynthetic algae. Production of these antivitamins by *M. aeruginosa* does not require co-culture, but putative biosynthesis genes are significantly upregulated when a competing algal species is present. Furthermore, we found that thiamin and methoxythiamin oxidation products, as well as one novel compound with structural similarity to bacimethrin, all accumulated to higher levels in the co-culture medium. Each inhibited the growth of wild-type *C. reinhardtii* when added individually with little or no effect on *M. aeruginosa*. Since the addition of thiamin largely eliminated the inhibitory effect of these compounds, it is likely that they all interfere with thiamin metabolism in *C. reinhardtii*. We also found that CC-25, a *C. reinhardtii* mutant unable to produce thiamin, is extremely sensitive to both bacimethrin and methoxythiamin. Also of note, our results may explain previous contradictory co-culture results: the green alga *Oocystis marsonii* was not inhibited by *M. aeruginosa* exudates when using WC medium (48) which contains thiamin, whereas allelopathic interactions were noted between *Chlorella vulgaris* and *M. aeruginosa* when BG-11 was used, and no thiamin addition was indicated (20).

It is possible that *M. aeruginosa* acquired the ability to synthesize bacimethrin from a bacterial source by horizontal gene transfer, a frequent occurrence in this species (49), or perhaps, given the lack of homology noted earlier, more likely the pathway evolved independently. The enzymes involved in bacimethrin biosynthesis are ubiquitous and may have been recruited from other pathways. It is, however, noteworthy that all the gene clusters examined in this study contain an ABC transport gene (Fig. 3B); ABC transporters are known to be involved in HMP transport (50) and are often found in gene clusters supporting the production and export of antibiotics (51). The thiaminase I gene, believed to be involved in extracellular degradation of thiamin and present in the previously published *C. botulinum* gene cluster (25) and other bacterial clusters (35), was not found in *M. aeruginosa*. Also noteworthy is the presence of ThiD2 in five of the six gene clusters. As previously mentioned, ThiD2 catalyzes the phosphorylation of HMP monophosphate, but not HMP or its toxic analogs (36). This potentially enables cells to discriminate between phosphorylated HMP and bacimethrin, which would make them less vulnerable to bacimethrin toxicity. We used InterPro (https://www.ebi.ac.uk/interpro/) to determine that ThiD2 (family: PF17792) is not present in *C. reinhardtii. C. reinhardtii* uses ThiD in thiamin biosynthesis (Fig. 4A), which has wide substrate specificity, enabling the production of methoxythiamin from bacimethrin and making it sensitive to bacimethrin as we have shown in this study. Less specificity has the positive side benefit of enabling the scavenging of thiamin degradation products (36).

We determined, for the first time, the presence of bacimethrin in a freshwater environment, Upper Klamath Lake, Oregon (Fig. 4E). This lake has an annually reoccurring *Microcystis* bloom that peaks in August, coincident with our sampling event (44–46). The concentration of bacimethrin in Upper Klamath Lake ranged from 3 to 8 pM and was roughly double at two sites during the algal bloom compared to pre-bloom, providing evidence that wild populations of *Microcystis* can produce bacimethrin. Although the observed bacimethrin concentrations are quite low, they are within the published range of freshwater concentrations (0.5–420 pM) and whole-cell uptake affinities (K_m_ 9.5 pM to 1.2 nM) of its non-toxic congener HMP (15, 22). Furthermore, the environmental bacimethrin concentrations are close to those that caused inhibition in the thiamin-requiring mutant CC-25 (Fig. 5A). Additional research will be required to definitively connect environmental bacimethrin production to *Microcystis* ecophysiology and determine the role that it and other antivitamins may play in structuring algal or perhaps even microbial communities.

Although allelopathy has been argued against as a cause of HABs in marine ecosystems (21), it is more likely to play a role in freshwater ecosystems where cell densities are typically higher and turbulence is lower. Previous research on such interactions between *M. aeruginosa* and other algae has primarily focused on the wide range of intracellular cyclopeptides produced by *M. aeruginosa* or lipophilic metabolites, although the exact mechanisms of inhibition and their environmental significance are not fully understood (20, 52, 53). Antimetabolites from cyanobacteria with known inhibitory targets, such as the non-proteinogenic amino acid β-methylamino-L-alanine (BMAA) (54) or the shikimate pathway inhibitor 7-deoxy-sedoheptulose (55), are consequential in the context of cumulative trophic toxicity and drug discovery, respectively, but they have not been shown to alter plankton composition. In contrast, thiamin antivitamins could effectively influence species composition because the concentrations of thiamin and its precursors can be extremely low in the water column (15), and high-affinity transporters are used in their acquisition (22, 23).

Given thiamin’s scarcity and its critical function in universal biochemical pathways, researchers have long sought to understand the influence of thiamin on aquatic ecosystems and primary productivity (11, 12, 56, 57), but none previously addressed the role of antivitamins. Algae like *C. reinhardtii,* which are able to synthesize thiamin (B_1_-prototrophs) but downregulate biosynthesis in the presence of exogenous thiamin (58), likely take up antivitamins and may tolerate low levels. However, based on our experiments with the thiamin-deficient mutant, organisms that are unable to synthesize thiamin (auxotrophs) will likely be very sensitive to antivitamins unless they have acquired resistance (59). Although a relatively low percentage of eukaryotic algae are auxotrophic (7), percentages are higher among members of the dinoflagellate, euglenoid, and cryptomonad phyla (9). Reduced biomass of cryptomonads has been reported during a HAB bloom along with that of green algae and diatoms (60). Competing species of cyanobacteria may not be susceptible to these antivitamins as freshwater cyanobacteria are generally prototrophic for thiamin (61), but a large percentage of marine bacterioplankton are auxotrophs for thiamin (8), and bacterial composition in freshwater ecosystems has been shown to be influenced by *Microcystis* blooms (62). Although we typically think of *M. aeruginosa* in competition for light and nutrients with other phototrophs, its use of urea likely leads to direct competition with heterotrophs (63).

These ecological findings, along with thiamin’s inherent instability in surface waters (including sensitivity to higher temperatures, 64), underscore the importance of gaining a deeper understanding of this phenomenon. Given the widespread prevalence and major impact of this toxin-producing organism on sensitive freshwater ecosystems worldwide, it is critical to understand its strategies for dominance. It is our hope that a better understanding of such mechanisms will lead to new strategies for the control and abatement of HABs.

## MATERIALS AND METHODS

### Alga strains and growth conditions

Wild-type *C. reinhardtii* (CC-125) and thiamin-requiring Thi-8 mutant (CC-25) were obtained from the Chlamydomonas Resource Center (University of Minnesota). *M. aeruginosa* PCC 7806 was purchased from the Pasteur Culture Collection (Paris, France). BG-11 (65) was used for experiments involving CC-125 and PCC 7806. BG-11 supplemented with 2% TAP and 1 nM thiamin were used for experiments involving CC-25. Further details of culture maintenance and experimental preparations are included in the Supplemental Methods.

### Microfluidic device

A microfluidic device with precise control of the chemical environment was used to measure the growth rate of both wild-type *C. reinhardtii* and CC-25 in an array of microhabitats (30). An agarose gel was molded with a silicon master and soaked in medium overnight. Cells are seeded onto the patterned gel, which was then secured in a Plexiglas manifold atop a glass slide. Arrays of microhabitats are flanked by side channels through which the source and sink medium are pumped with a syringe pump (Fig. 1C). Molecular diffusion through the agarose gel establishes a linear gradient of chemicals included in the source channel flow but is absent from the sink channel (Fig. 1C). Details are included in Supplemental Methods.

### Preparation of exometabolome extracts

*C. reinhardtii*, *M. aeruginosa*, and co-cultured cells were grown in BG-11 for 14 days to generate a spent medium. A 0.2 µm syringe filter was used to remove cells and other debris. The filtrate was lyophilized in 50 mL polypropylene tubes, and cellular metabolites were resuspended with 1 mL of methanol (MeOH).

### Chemical syntheses

Methoxythiamin sulfate synthesis, based on a previous report (37) with some modifications, is shown in Fig. S6A. Methoxythiochrome synthesis is shown in Fig. S6B. Details are included in Supplemental Methods.

### LC-MS analyses

For untargeted analyses, exometabolome extracts were analyzed by ultra-high-performance liquid chromatography (U-HPLC, Thermo Vanquish) coupled to a quadrupole mass spectrometer QE-HF (Thermo) with an electrospray ionization (ESI) source in positive ion mode. For targeted analyses, the same HPLC was coupled to an Orbitrap QE-HF operated in positive ion mode using data-dependent acquisition (DDA) and parallel reaction monitoring (PRM). The targeted CMT measurements were conducted on an Exion LC coupled with an X500B Q-TOF system and were verified with a commercial standard (Alfa Chemistry, Protheragen Inc., Ronkonkoma, NY). Details are included in Supplemental Methods.

### Exometabolome analysis

Orthogonal Projections to Latent Structures Discriminant Analysis (OPLS-DA) was used to analyze differences in the chemical composition of the exometabolomes obtained via untargeted analysis (*n* = 3 for each). Details of the OPLS-DA analysis are in Supplemental Methods. Volcano plots were generated using GraphPad Prism 9. Compounds scoring below a threshold of *P* < 0.05 and exceeding a 2-fold change either up or down (Log_2_ >1 or Log_2_ <1) were classified as significantly higher or lower respectively relative to the comparison exometabolome.

### RNA extraction and RT-qPCR

RNA extraction from *M. aeruginosa* cells, cDNA synthesis, and RT-qPCR were done using standard protocols. Details are included in Supplemental Methods.

### Environmental bacimethrin analysis

Samples for bacimethrin analysis were collected from two sites in Upper Klamath Lake (Oregon) using techniques previously described (13, 15). Sampling was conducted in May and August 2023 to capture the pre- and peak-bloom periods, respectively. Details are included in Supplemental Methods.

## Data Availability

All data generated for this paper are included in the figures, supplemental figures, or supplemental data file (metabolomic data). The LCMS data shown in the supplemental figures have been deposited to the public repository MetaboLights (ftp://massive-ftp.ucsd.edu/v09/MSV000098018/).

## SUPPLEMENTAL MATERIAL

Data set S1 mbio.01608-25-s0001.xlsx Untargeted metabolomic data.

Supplemental Figures and Table mbio.01608-25-s0002.pdf Figures S1-S13; Table S1.

Supplemental Methods mbio.01608-25-s0003.docx All methodological details not included in the text.

ASM does not own the copyrights to Supplemental Material that may be linked to, or accessed through, an article. The authors have granted ASM a non-exclusive, world-wide license to publish the Supplemental Material files. Please contact the corresponding author directly for reuse.
