# Supplementary material for: Harmful algal bloom species Microcystis aeruginosa releases thiamin antivitamins to suppress competitors
Source: mBio. 2025 Jul 2;16(8):e01608-25. doi: 10.1128/mbio.01608-25 (PMC12345177; doi:10.1128/mbio.01608-25)
Supplement: Supplemental Figures and Table — Figures S1-S13; Table S1. [file mbio.01608-25-s0002.pdf]

Supplementary Materials

(13 figures and 1 table)

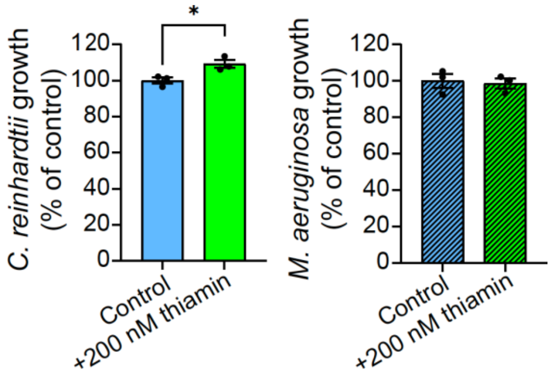

Fig. S1. Thiamin addition is not inhibitory of *C. reinhardtii* and *M. aeruginosa* growth. Thiamin (200 nM) was added to the growth medium of both *C. reinhardtii* and *M. aeruginosa*. No adverse effect on growth rate was observed.

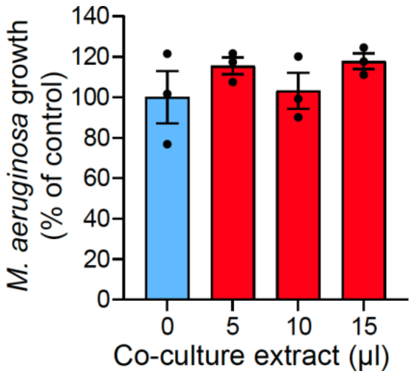

Fig. S2. Methanol extract of co-culture spent medium does not inhibit *M. aeruginosa* growth. Relative growth rate of *M. aeruginosa* with methanol extract of co-culture medium added to growth medium.

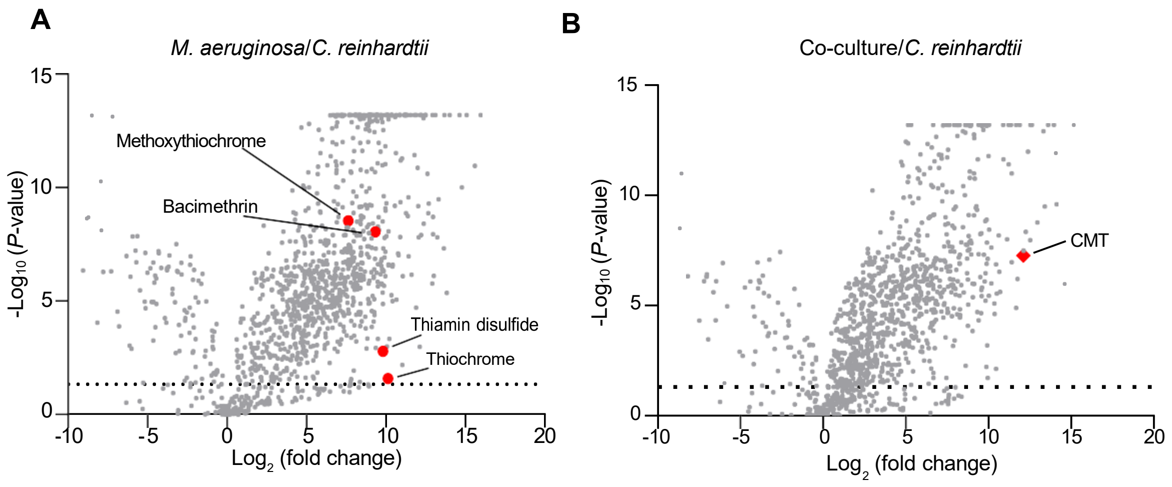

Fig. S3. Thiamin antivitamin levels are many-fold higher in *M. aeruginosa* single culture and co-culture extracts compared to *C. reinhardtii* extracts. (A) Volcano plot of compounds found by LC-MS in *M. aeruginosa* extracts relative to *C. reinhardtii*. Thiamin antivitamins are shown as labeled red dots. (B) Volcano plot of compounds found by LC-MS in co-culture extracts relative to *C. reinhardtii.* CMT (4-cyclopropyl-6-methoxy-1,3,5-triazin-2-amine) is shown as labeled red diamond. In (A) and (B) dotted line: P = 0.05.

MS analysis of the thiamin analogs

Name	Formula	*m/z*
Bacimethrin	$C_6H_9N_3O_2$	156.0769
2'-Methoxythiamin	$C_{12}H_{16}N_4O_2S$	281.1072
Thiochrome	$C_{12}H_{14}N_4OS$	263.0959
Methoxythiochrome	$C_{12}H_{14}N_4O_2S$	279.0908
Thiamin disulfide	$C_{24}H_{34}N_8O_4S_2$	282.1143

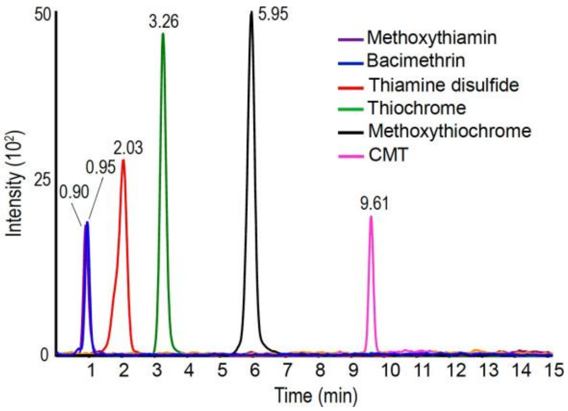

Fig. S4. LC-MS/MS analysis of commercial and synthesized thiamin antivitamins. Names, formulas, and m/z of standards used. Composite chromatograms (XIC of PRM-HR) of the antivitamin standards labeled with retention time. Peaks shown were obtained by loading 40 pmol methoxythiamin, 1 pmol bacimethrin, 10 pmol thiamine sulfide, 5 pmol thiochrome, 15 pmol methoxythiochrome and 2 pmol CMT on a C18 column.

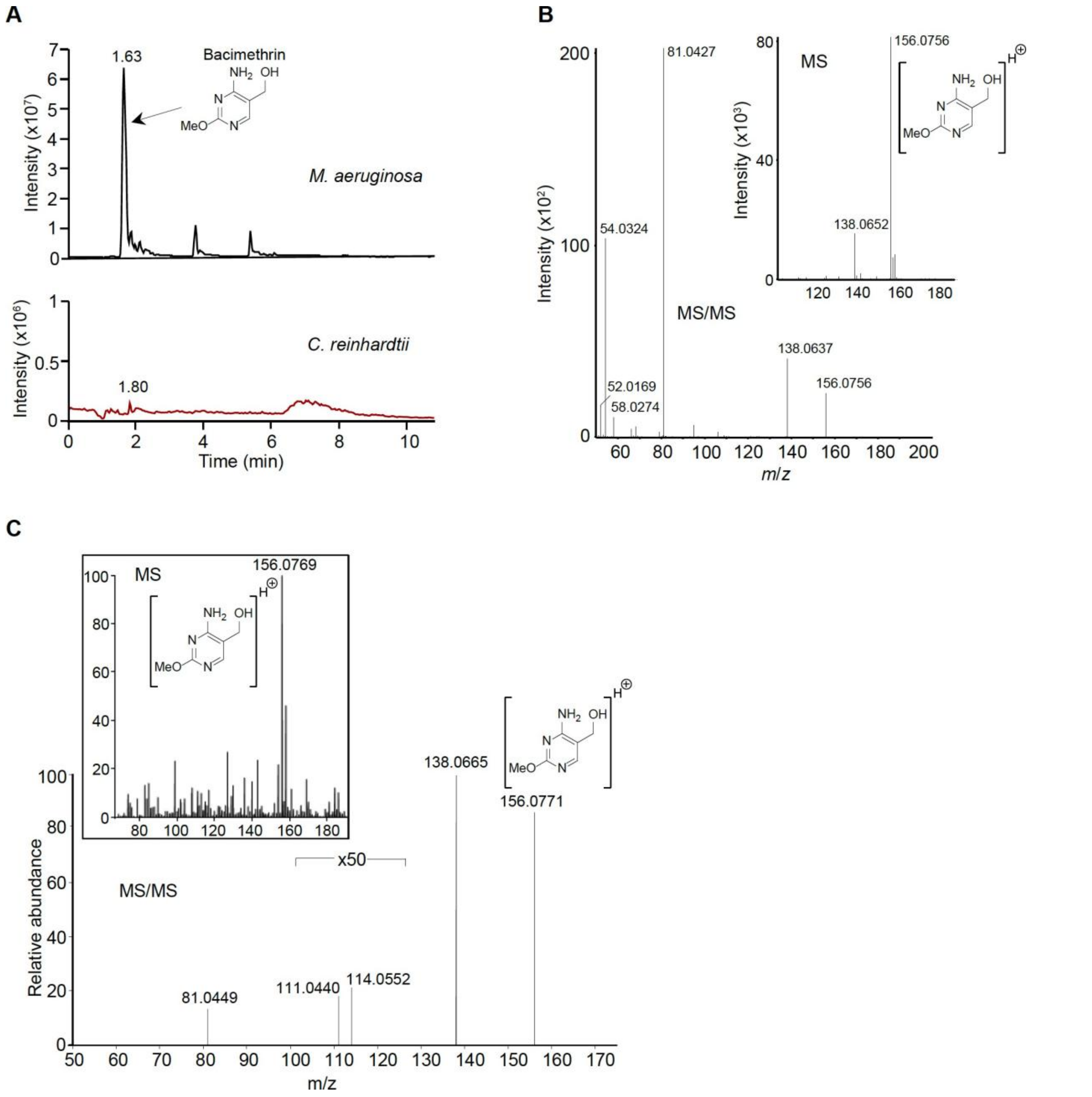

Fig. S5. Presence of bacimethrin in *M. aeruginosa* extract is confirmed with LC-MS/MS. (**A**) Extracted Ion Chromatograms (XIC) of bacimethrin (m/z 156.0769) in *M. aeruginosa* and *C. reinhardtii* single culture extracts obtained by LC-MS/MS. (**B**) MS (inset) and MS/MS spectra of bacimethrin standard. **(C)** MS (inset) and MS/MS spectra of bacimethrin in *M. aeruginosa* single culture extract.

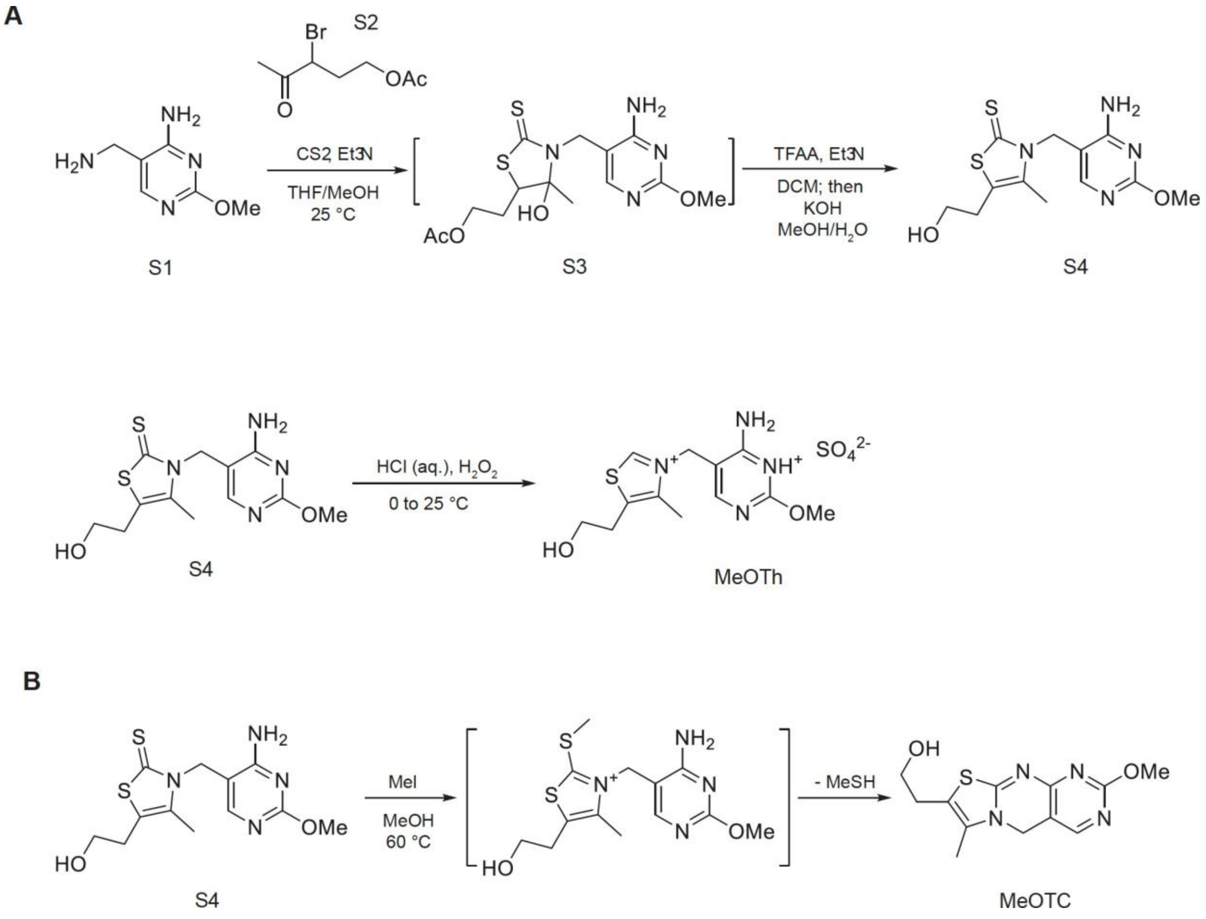

Fig. S6. Chemical syntheses of 2′-methoxythiamin (A) and methoxythiochrome (B).

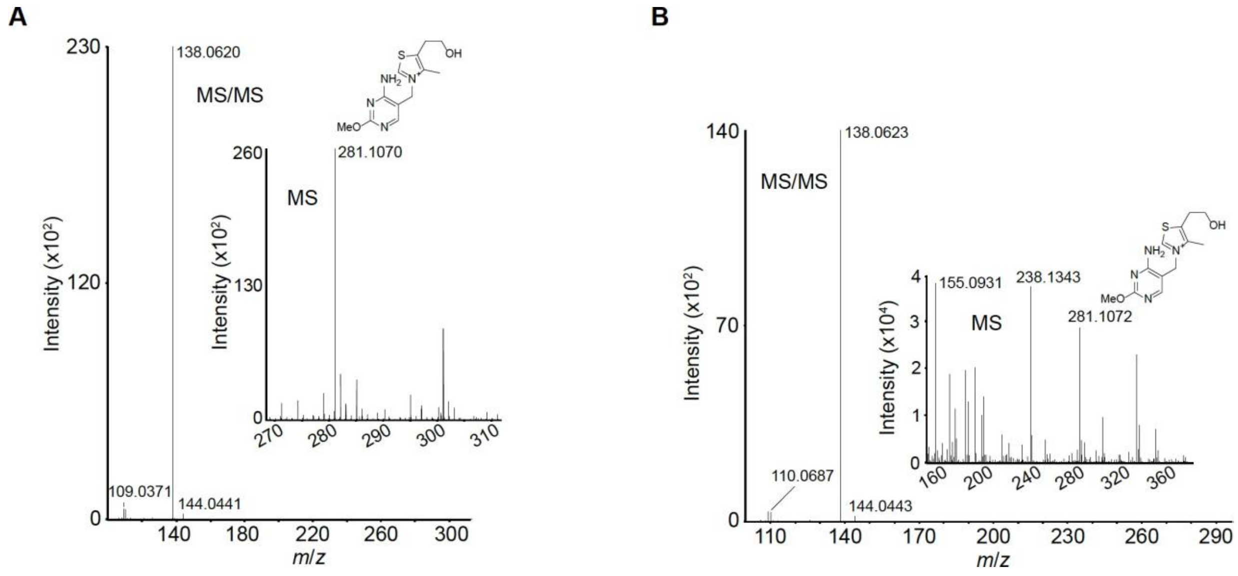

Fig. S7. **Presence of methoxythiamin in *M. aeruginosa* extract is confirmed with by LC-MS/MS. (A)** MS (inset) and MS/MS spectra of methoxythiamin standard. **(B)** MS (inset) and MS/MS spectra of methoxythiamin in *M. aeruginosa* single culture extract. The MS/MS spectrum of m/z 281.106 for methoxythiamin was acquired using a relatively low collisional energy (CE = 15). The predominant fragment ion at m/z 138.065 was accompanied by several fragment ions with relatively low signal intensity.

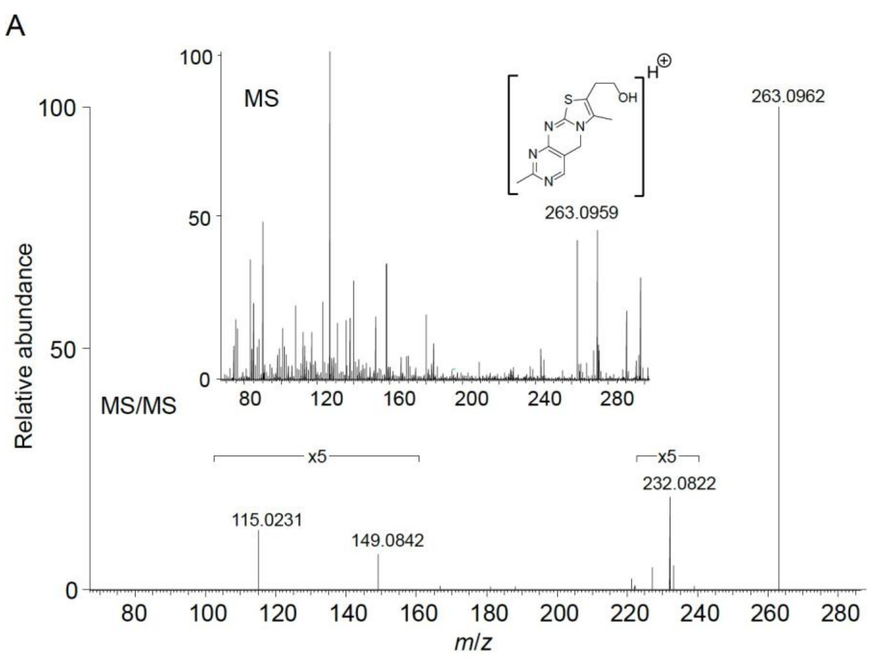

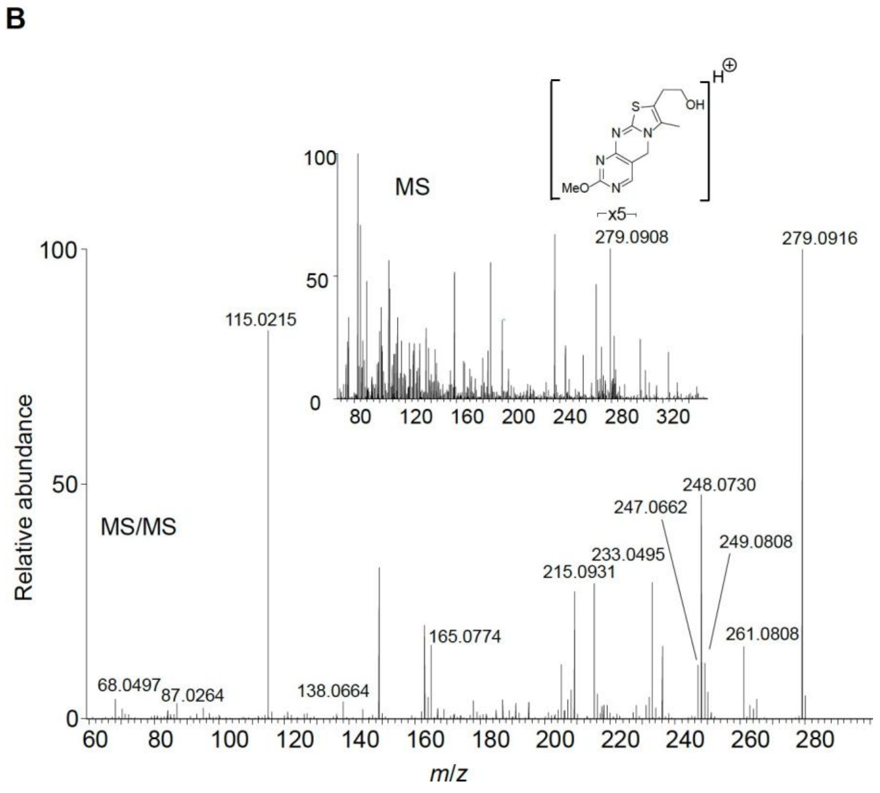

Fig. S8. LC-MS/MS spectra for thiochrome (A)and methoxythiochrome (B). The chemical structure of the parent product is labeled on the MS spectra (inset) with the MS/MS spectrum.

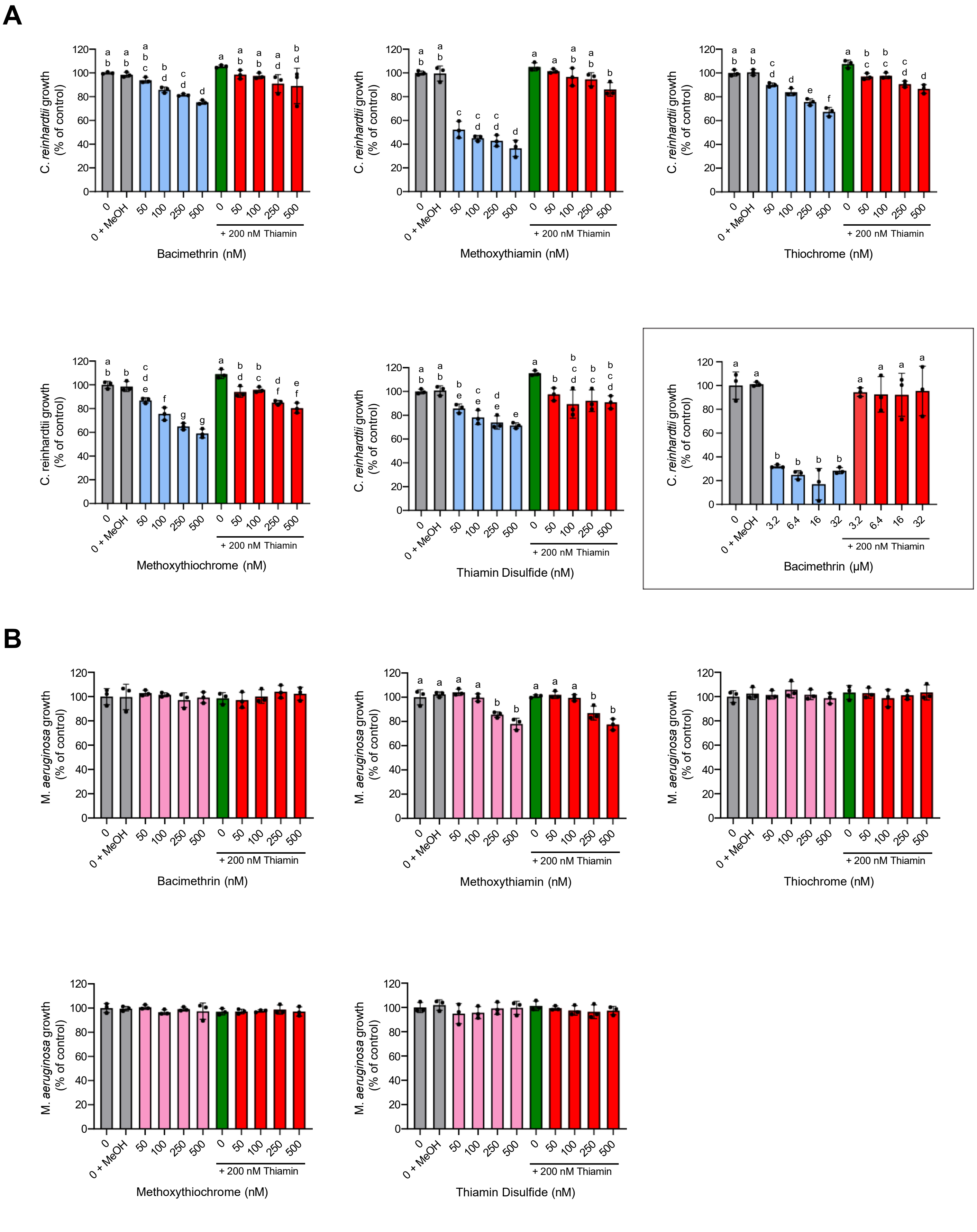

Fig. S9 (caption on following page)

Fig. S9. Thiamin antivitamins and their oxidation products cause thiamin deficiency in *C. reinhardtii* but not *M. aeruginosa*. (A) Relative growth rate of *C. reinhardtii* (relative to control with no added MeOH) with individual thiamin antivitamins added separately (blue bars) and added with 200 nM thiamin (green and red bars). Figure in box shows relative growth rate of *C. reinhardtii* with higher concentrations (µM) of bacimethrin. **(B)** Relative growth rate of *M. aeruginosa* (relative to control with no added MeOH) with individual thiamin antivitamins added separately (striated blue bars) and added with 200 nM thiamin (striated green and red bars). In (A) and (B) the volume of methanol (MeOH) added to the +MeOH control was equivalent to the highest volume of stock solution added. In (A) and (B) data are means ± SEM, with $n = 3$. One-way ANOVA was performed to show the significance of differences between data sets $P < 0.001$, with letters indicating statistical significance from a Tukey post-hoc test.

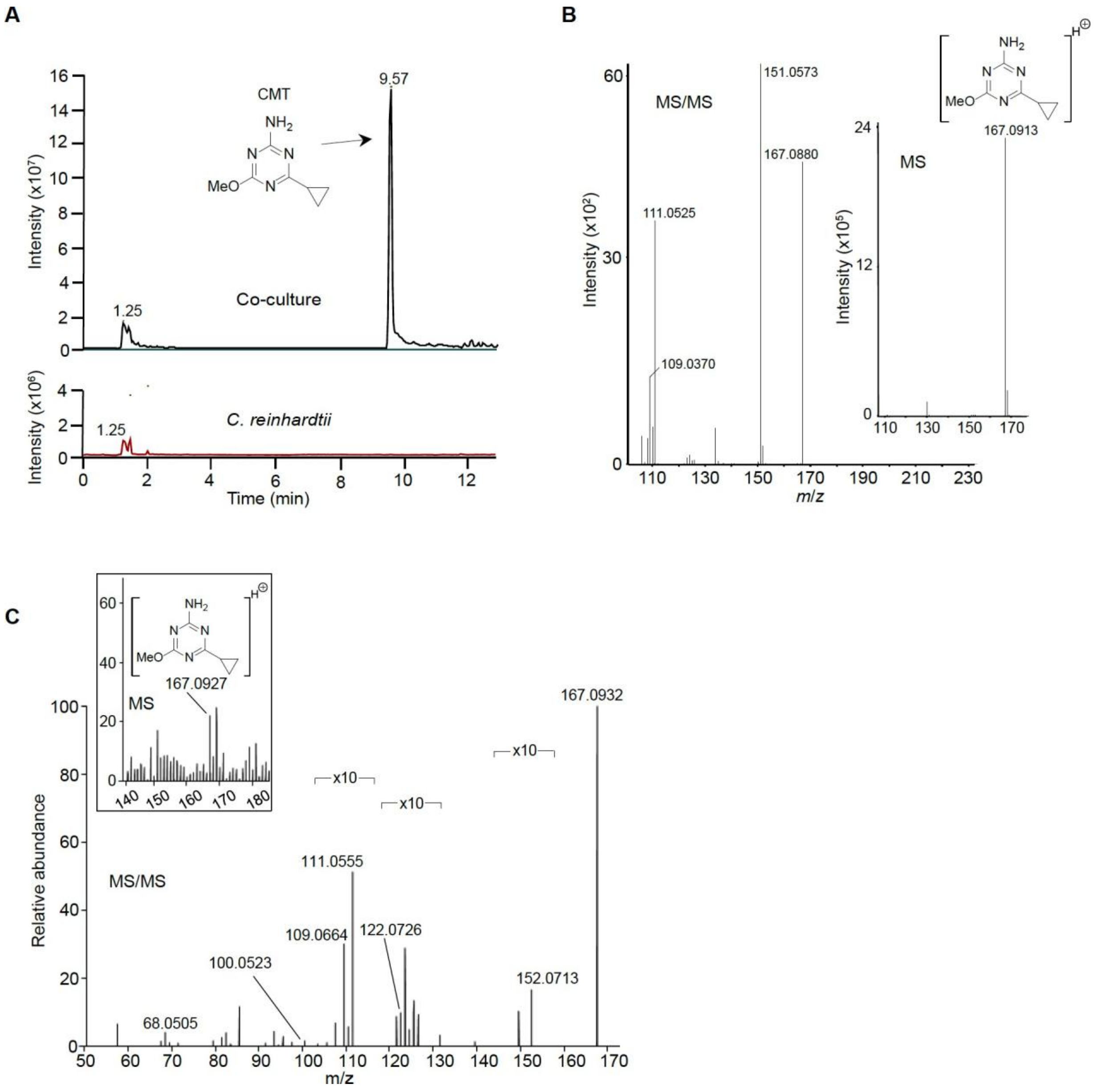

Fig. S10. Presence of CMT in *M. aeruginosa* extract is confirmed with LC-MS/MS. (A) Extracted Ion Chromatograms (XIC) of CMT (m/z 167.0927) in co-culture and *C. reinhardtii* single culture extracts obtained by LC-MS/MS. (**B**) MS (inset) and MS/MS of CMT standard **(C)** MS (inset) and MS/MS of CMT in *M. aeruginosa* single culture extract.

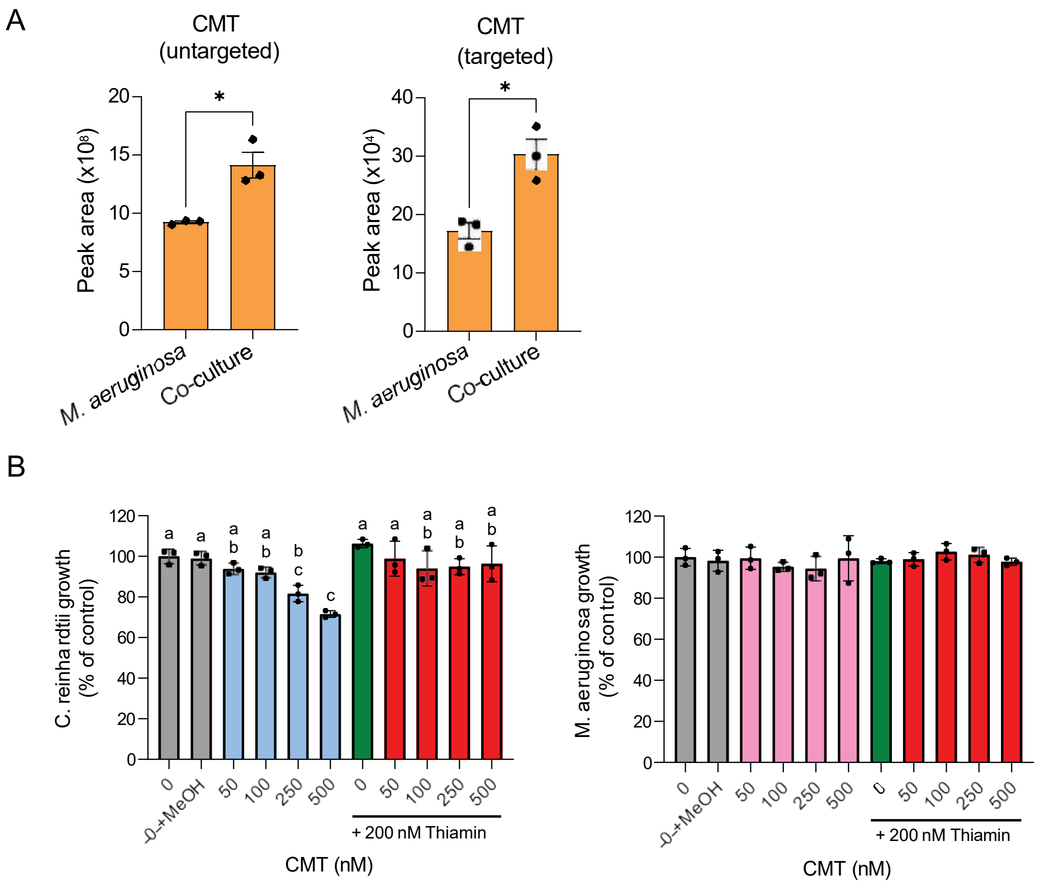

Fig. S11 HMP-like analog CMT is elevated in co-culture exometabolome and causes thiamin deficiency in C. reinhardtii but not M. aeruginosa. (A) Levels of CMT in M. aeruginosa single culture and co-culture extracts obtained by nontargeted (left) and MRM LCMS/MS (right). (B) Relative growth rate of C. reinhardtii (blue bars) and M. aeruginosa (bluestriated bars) with added CMT. Green and red bars show relative growth rates with 200 nM thiamin added. Rates are relative to control with no added MeOH. The volume of MeOH added to the +MeOH control was equivalent to the highest volume of stock solution added. In (A) and (B) data are means ± SEM, with n = 3. In (A) Student's t test was carried out to show the significance of differences between data sets; *P < 0.02, **P < 0.01, ***P < 0.001, ****P < 0.0001. In B, one-way ANOVA was performed to show the significance of differences between data sets P < 0.002, with letters indicating statistical significance from a Tukey post-hoc test.

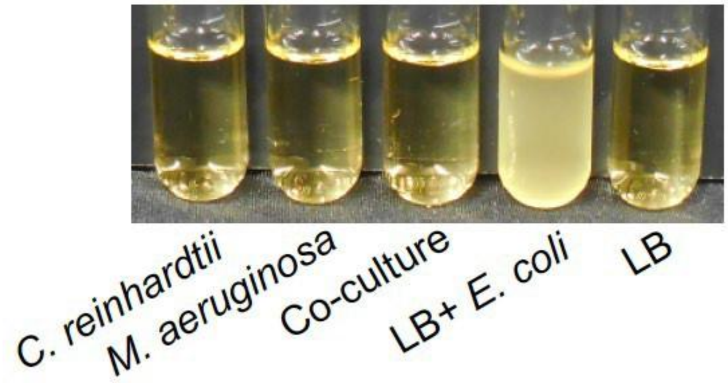

Fig. S12. Visual confirmation that algal cultures are not contaminated with bacteria. A small volume (20 µl) of cell-free medium from individual cultures and co-culture was added to bacterial growth medium (LB medium) in glass culture tubes. Tubes were incubated overnight in a 37 ˚C shaker. LB medium showed no signs of bacterial growth unless inoculated with *E. coli*.

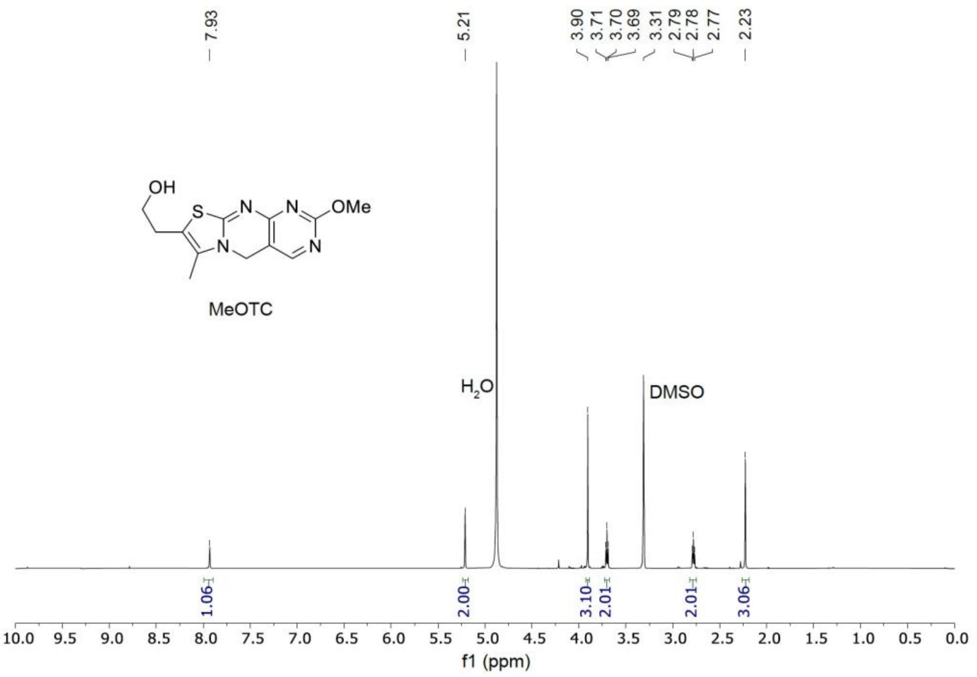

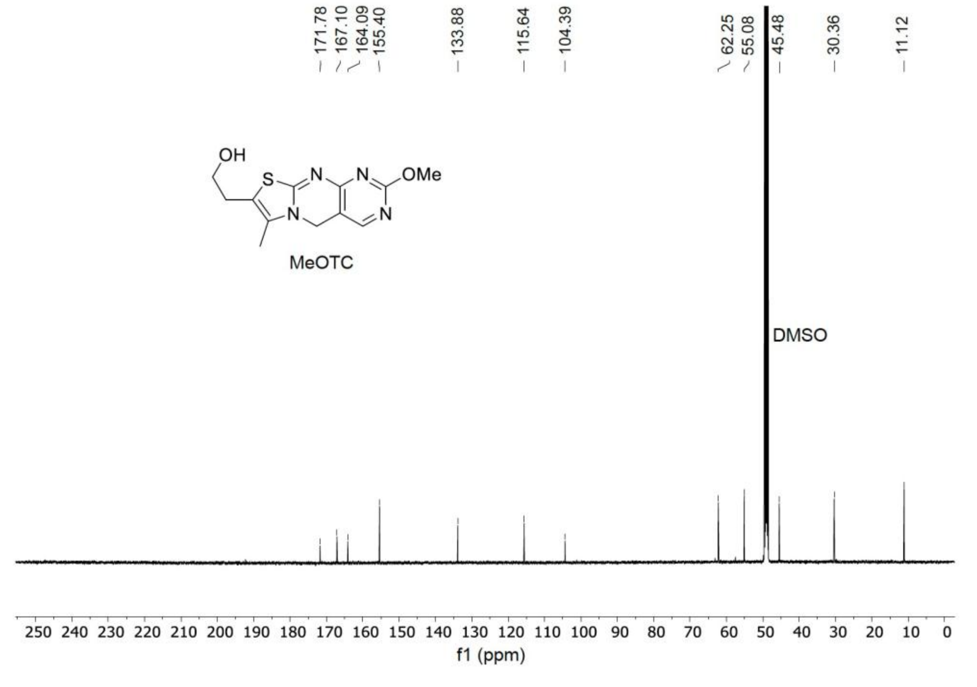

Fig. S13. NMR spectra of methoxythiochrome (MeOTC). ^{1}H NMR of MeOTC was measured at 500 MHz in deuterated methanol (CD_3OD) (top) and ^{13}C NMR of MeOTC was measured at 125 MHz also in CD_3OD (bottom).

Table S1: Primers used in this study for qRT-PCR of the bacimethrin biosynthetic genes in *M. aeruginosa*

Thymidylate synthase-F: TTGCCGAAACCTGGGTAAA
Thymidylate synthase-R: CGACGGCCATTAGGTCAATTA
Methyltransferase-F: GGTTGGATCTCTCTCCCTACTA
Methyltransferase-R: CGTAGGACTGACTGGGTAATTG
16S rRNA-F: GGGTGAGTAACGCGTAAGAA
16S rRNA-R: GCTCTTCTCCAGGCCAATTA
