# Supplementary material for: Harmful algal bloom species Microcystis aeruginosa releases thiamin antivitamins to suppress competitors
Source: mBio. 2025 Jul 2;16(8):e01608-25. doi: 10.1128/mbio.01608-25 (PMC12345177; doi:10.1128/mbio.01608-25)
Supplement: Supplemental Methods — All methodological details not included in the text. [file mbio.01608-25-s0003.docx]

**Supplemental Materials: Supplemental methods with details needed to replicate experimental conditions.**

### **Algal Growth Conditions and Methods.** All cultures (CC-125, wildtype *C. reinhardtii*, CC-25, thiamin-requiring Thi-8 mutant, and *M. aeruginosa* PCC 7806) were kept at 25 °C with 16 µmol m^-2^ s^-1^ continuous light unless otherwise stated. Batch cultures were placed on a shaker (130 rpm) in glass flasks (50 mL) or 96-well plates (300 µL). All experiments were initiated with exponential growth phase cells. Co-culture experiments were inoculated at cell densities of approximately 1 x 10^6^ cell mL^-1^ and 2 x 10^5^ cell mL^-1^ for *M. aeruginosa* and *C. reinhardtii* respectively. CC-25 cells were transferred twice into thiamin-free medium prior to experimentation to generate thiamin-limited cells for experiments. Cultures were received axenic from the Pasteur Institute, and we further confirmed that strains were axenic by periodic light microscopic examination of cells from stock cultures under a 100 x magnification, which enables visualization of bacteria and differentiation of cyanobacteria by their blue-green color. We also added 20 µL of spent medium from batch cultures/co-culture experiments each to Luria-Bertani (LB) bacterial medium; no bacterial growth was observed after 24 h (Fig. S12). Algal and cyanobacterial cell growth was monitored either by measuring cell density using a hemocytometer or chlorophyll *a* absorbance at 665 nm using a microplate reader (Power Wave XS, BioTek, USA). To minimize contamination in our exometabolomes, glass culture flasks were baked in a muffle furnace at 500 °C for 5 hours prior to use.

**Batch Growth Inhibition Experiments.** Wildtype *C. reinhardtii* and *M. aeruginosa* were grown into 96-well plates with additions of bacimethrin, methoxythiamin, thiochrome, methoxythiochrome, thiamin disulfide and CMT (0.10 - 500 nM) from stock solutions in MeOH. CC-25 was grown in 96-well plates with additions of methoxythiamin (10 -100 pM). Each was treatment was repeated with excess thiamin (200 nM) or less as specified in captions. Algal cell density was monitored daily with absorbance, and growth rate was calculated for each triplicate.

**Microfluidic Device.** As described in Kim et al. (1), algae were grown in microhabitats with defined chemical environments. Briefly, a silicon master with designed patterns was fabricated using SU-8 negative resist photolithography. A 3% agarose gel membrane was molded out of the silicon master and soaked in cell culture medium overnight before device assembly. For device assembly, 200 μL of *C. reinhardtii* cell culture at exponential growth phase (~3 x 10^6^ cells mL^-1^) was first seeded onto the patterned agarose gel with four parallel sets of array microhabitats. The gel was then sandwiched between a Plexiglas manifold and a glass slide and screwed down to a stainless-steel frame to prevent leakage. Each set consists of a 4-by-4 array of 200 (L) × 200 (W) × 100 μm (H) microhabitats and two 400 (W) × 200 μm (H) side channels.

The flow through the side channels was controlled by a syringe pump (KDS230, KD Scientific, Holliston, MA) together with 10 mL syringes (BD, Franklin Lakes, NJ) and medical grade tubing (ID = 0.25 mm, PharMed BPT, Cole-Parmer, Vernon Hills, IL) which connected to the inlets of the microfluidic device using cut gel-loading tips. A flow rate of 0.7 μL min^-1^ was maintained throughout the experiment. The assembled device and the flow control unit were kept in an incubator at 25°C under 12 μmol m^-2^ s^-1^ illumination using fluorescent light bulbs for spent medium experiments (4200K, Lights of America; 3500K, SLI Lighting E-LITE) and under 100 μmol m^-2^ s^-1^ (Quantum Board, Horticultural Lighting Group) for the methoxythiamin exposure experiements. The entire setup was taken to the microscope for imaging once every day. Images were taken with an epi-fluorescence microscope (Olympus IX51, Center Valley, CA) equipped with a CCD camera (Cascade 512B, Photometrics, Tucson, AZ) and image acquisition software (IPLab imaging software, BD) was used to enumerate cell density. When exposing the cells to methoxythiamin (Fig. 4*C* and Fig. 5*C*), the source medium containing methoxythiamin (ranging from10 pM up to 50 nM) was replaced daily to reduce the impact of methoxythiamin degradation.

**Chemical Synthesis of Methoxythiamin (MeOTh) and Methoxythiochrome (MeOTC).** Solvents for air sensitive reactions were purchased from Fisher, sparged with ultrahigh purity (UHP) grade nitrogen, and either passed through two columns containing reduced copper (Q-5) and alumina (THF) or passed through two columns of alumina (CH_2_Cl_2_) prior to use. All other chemicals and reagents were purchased from commercial sources (Sigma-Aldrich, Oakwood Chemical, Strem, TCI, Alfa Aesar, Acros, and Fisher) and used without further purification. All manipulations of air and water sensitive compounds were carried out under nitrogen by using standard Schlenk line technique. Flash column chromatography with silica gel (particle size 40–64 μm, 230-400 mesh) was used to purify final products. ^1^H and ^13^C NMR spectra were recorded on Bruker AVANCE III HD (^1^H, 400 MHz) spectrometer with a BBF/1 H broadband observe probe or Bruker AVANCE III HD (^1^H, 500 MHz) spectrometer with a broadband Prodigy cryoprobe. All the NMR spectra were processed with MestReNova software. Chemical shifts (δ) for ^1^H NMR spectra were referenced to protons on the residual solvents (7.26 ppm for CDCl_3_, 3.31 ppm for CD_3_OD). Chemical shifts (δ) for ^13^C NMR spectra were referenced to the deuterated solvents themselves (77.16 ppm for CDCl_3_, 49.00 ppm for CD_3_OD). NMR spectroscopic data were reported as follows: chemical shift, multiplicity (s = singlet, d = doublet, t = triplet, br = broad), integration and coupling constants (Hz). High resolution mass spectrometry (HRMS) analyses were performed on a Thermo Scientific Exactive Orbitrap MS system equipped with an Ion Sense DART ion source.

MeOTh sulfate synthesis is shown in Fig. S6*A* and was based on previous report (2) with some modifications. In brief, for S4 synthesis, Et_3_N (0.04 ml, 0.28 mmol, 2.0 equiv) and CS_2_ (0.1 mL) was added to a solution of S1 (21 mg, 0.14 mmol, 1.0 equiv) in a mixture of THF (1 mL) and MeOH (0.5 ml) at 25 °C. After stirring for 10 min, a solution of S2(3, 4) (32 mg, 0.14 mmol, 1.0 equiv) in THF (0.5 ml) was added at 25 °C. The reaction mixture was further stirred for 30 min, then the solvent was removed under vacuum. The residual was redissolved in DCM (1 mL), and TFAA (0.03 mL, 0.20 mmol, 1.0 equiv) and Et_3_N (0.06 ml, 0.41 mmol, 3.0 equiv) was added at 25 °C. The reaction was further stirred for 1 h, then the solvent was removed under vacuum. The residual was redissolved in MeOH (1 mL), and 1 M aqueous KOH solution (1 mL) was added at 25 °C. The reaction was further stirred for 1 h, then diluted by water (10 mL) and further extracted with EtOAc (10 mL × 3). The organic phase was dried with Na_2_SO_4_, filtered, and concentrated under reduced pressure. The residual was purified by flash chromatography (DCM/MeOH = 40/1 to 20/1) to give S4 (20 mg, 47% yield) as a light-yellow solid. All the spectroscopic data matched with the literature report (2). For synthesis of MeOTh, a solution of aqueous H_2_O_2_ (1.0 M, 0.048 ml, 0.048 mmol, 3.0 equiv) was added to a suspension of S4 (5.0 mg, 0.016 mmol, 1.0 equiv) in aqueous HCl (0.27 M, 0.40 mL) at 0 °C. The reaction was stirred at 25 °C for 6 h and evaporated to dryness to give MeOTh (5.0 mg, 83% yield) as a white solid. All the spectroscopic data matched with the literature report (2).

For synthesis of MeOTC (Fig. S6*B*), MeI (0.17 mL, 2.7 mmol, 27 equiv) was added to a solution of S4 (31 mg, 0.10 mmol, 1.0 equiv) in MeOH (4 mL). After stirring at 60 °C for 4 h, the reaction mixture was cooled and all the volatiles were removed under reduced pressure. The residual was mixed with saturated aqueous NaHCO_3_ (5 mL) and extracted with DCM (10 mL × 3). The organic phase was dried with Na_2_SO_4_, filtered, and concentrated under reduced pressure. The remaining solid was washed with minimal amount of DCM to give MeOTC (7.5 mg, 27% yield) as a light-yellow solid. ^1^H and ^13^C NMR spectra of MeOTC dissolved in deuterated methanol (CD_3_OD) are shown in Fig. S13. MeOTC: ^1^H NMR (500 MHz, CD_3_OD) δ 7.93 (s, 1 H), 5.21 (s, 2 H), 3.90 (s, 3 H), 3.70 (t, *J* = 6.0 Hz, 2 H), 3.31 (s, 3 H), 2.78 (t, *J* = 5.9 Hz, 2 H), 2.23 (s, 3 H). ^13^C NMR (125 MHz, CD_3_OD) δ 171.78, 167.10, 164.09, 155.40, 133.88, 115.64, 104.39, 62.25, 55.08, 45.48, 30.36, 11.12. HRMS (DART-MS): m/z calculated for C_12_H_14_N_4_O_2_S^+^ [M+H^+^] 279.0911, found 279.0947.

**LC MS Analyses of Methanol-Solubilized Exometabolomes.** For untargeted analyses, lyophilized-algal medium methanol extracts were analyzed by ultra-high-performance liquid chromatography (Thermo Vanquish, UHPLC) coupled to a quadrupole mass spectrometer QE-HF (Thermo) operated with a electrospray ionization (ESI) source in both positive and negative ion mode. Positive ion mode was determined to be more effective for the compounds of interest. HPLC was performed with a Accucore Vanquish C18+ column (1.5 µm, 2.1 x 100 mm) heated to 30 °C using an injection volume of 2 µL. The metabolites were eluted via solute gradient at 200 µL min^-1^ using 0.1% formic acid in water (solvent A) and 0.1% formic acid in acetonitrile, ACN, (solvent B). The gradient was as follows: 0–2.0 min (0% B), 2.0–4 min (0–15% B), 4-14 min (15-32% B), 14-19 min (32-50% B), 19-19.1min (50-100% B), 19.1-21 min (100% B), 21-21.1 min (100-0% B), 21.1-23 min (0% B). Standards of bacimethrin (AdipoGen, San Diego, CA), methoxythiamin (synthesized as described), thiochrome (Sigma-Aldrich), methoxythiochrome (synthesized as described), thiamin disulfide (TCI America, Portland, OR), and 4-cyclopropyl-6-methoxy-1,3,5-triazin-2-amine (CMT, Alfa Chemistry, Protheragen Inc., Ronkonkoma, NY) were also run to confirm retention times observed in untargeted analysis. MS conditions were as follows: ESI voltage 3.5 kV, sheath, aux, sweep gas flow rates: 50, 10, 1 (arbitrary units), capillary temp. 275 °C, aux gas heater 375 °C, S-Lens RF level 55%. Software packages Compound Discovered 3.2 and Xcalibur 4.3, and Mass Frontier 8.0 were used for MS data analysis and prediction of in silico fragments.

For targeted analyses of the same extracts, the same HPLC and column was used at 55°C using an injection volume of 3 µL for samples and standards. Samples were diluted to 20% MeOH in 0.1% formic acid prior to injection to improve retention times; standards were prepared in same solution. The metabolites were eluted via solute gradient at 250 µL min^-1^ using 0.1% formic acid in water (solvent A) and 0.1% formic acid in ACN (solvent B). The gradient was as follows: 0–4.0 min (0.5- 1.0% B), 4.0–8.5 min (1.0–20% B), 8.5-13.5 min (20-95% B), 13.5-15.5 min (95-99% B), 15.5-18 min (99-100% B), 18-19 min (100-0.5% B), 19- 25 min (0.5% B). The HPLC was coupled to an Orbitrap QE-HF operated under positive ion data dependent acquisition (DDA) and parallel reaction monitoring (PRM) mode with targeted m/z for 5 standard precursors in the inclusion list. MS conditions were as follows: ESI voltage 3.8 kV, sheath, aux, and sweep gas flow rates: 20, 7, 1 (arbitrary unit), capillary temperature. 320 °C, aux gas heater 250 °C, S-Lens RF level 60. A sum of 5 to 7 product ions from each precursor with mass tolerance at 10 ppm was used to generate the XIC chromatograms in PRM data quantitative analysis by Xcalibur 4.3 software.

For the targeted CMT data included in the paper (Fig. S11*A*), the analysis was conducted on an Exion LC coupled with X500B Q-TOF system. A Luna C18 column from Phenomenex (3 µm, 2.0 x 100 mm) was used at a flow rate of 200 µL min^-1^, temp 30 °C. Solvents: A) 0.1% formic acid; B) 95% acetonitrile with 0.1% formic acid. A/B gradient: 0-5 min (5% B), 5-10 min (40% B), 10-13 min (90% B), 13-14 min (90% B), 14-15 min (5% B), 15-20 min (5% B). Injection volume: 2 µL for standards and 5 µL for samples, Loop volume 50 µL. MS analysis: Sciex X500B operated in ESI positive ion TOF mode. Calibration was carried out with negative calibrant using CDS system (ESI voltage: 5.5 kV; ion source gas1 and 2: 20 psi; curtain gas: 25 (arbitrary unit); CAD gas: 7 (arbitrary unit); source temperature: 350 °C; DP: 80V; accumulation time: 0.25 s; scan: an MS full scan from *m/z* 100 to *m/z* 1000 in profile mode followed by MRM HR scan acquired from 0 min to 20 min at CE 10V.

Sciex OS 2.0, was used for all data processing of targeted raw files generated in X500B, XIC chromatograms of five standards with mass tolerance at 0.02 Da from MRM HR runs are shown in Fig. S4.

**Exometabolome Analysis.** Orthogonal Projections to Latent Structures Discriminant Analysis (OPLS-DA) was used to analyze differences in the chemical composition of the exometabolomes using the *C. reinhardtii* data set as the control. The score plot for components t[1] and t[2] showed low variation among biological replicates (n = 3) and confirmed significant differences between the three separate algal exometabolomes (Fig. 2*B*). The two sample sets that include *M. aeruginosa* exudates scored negative values relative to t[1], but were distinct from each other along the t[2] axis, whereas the samples containing *C. reinhardtii* exudates were negative on the t[2] axis and distinct from each other along the t[1] axis. Axis values of t[1] and t[2] shown corrected by a factor of 1.00001. The R2X[1] was 0.753 and the R2X[2] was 0.188. The ellipse shown in the figure represents Hotelling’s T2 95% confidence range (Fig. 2*B*).

**RNA Extraction and RT-qPCR.**  *M. aeruginosa* cells were pelleted by centrifugation from single cultures and co-cultures. RNA from triplicate pellets was extracted using the RNeasy Plant Mini Kit (Qiagen, Carlsbad, CA, USA). cDNA was synthesized from RNA using iScript^TM^ cDNA Synthesis Kit (Bio-Rad). Real-time qPCR was performed using gene specific primers (Table S1) and SYBR Green Supermix (Bio-Rad) in a CFX96 Real-Time PCR system (Bio-Rad). The *M. aeruginosa 16S rRNA* gene was used as internal control and relative changes in expression were analysed using the 2^-ΔΔCT^ method (5).

**Environmental Bacimethrin Analysis.** Samples for dissolved bacimethrin were collected from two sites in Upper Klamath Lake, Oregon, using techniques previously been described (6, 7); the southern site was offshore of Hanks Marsh (42.31046N, 121.84369W) and the northern site was at the mouth of the Williamson River (42.46121N, 121.96013W). Sampling was conducted in May and August 2023 to capture the pre- and peak- bloom periods, respectively. All sampling equipment was cleaned using 1 M hydrochloric acid and methanol as has previously been described (7). Samples were collected by boat from a depth of approximately 0.5 m using 1 L amber HDPE bottles (Nalgene). Samples were then prefiltered using a 100 micron mesh filter to remove metazoans, stored on ice, and transported back to shore for further processing. Gentle peristaltic filtration across a 0.2 micron Sterivex filter (PES membrane, Millipore, Burlington, MA, USA) was used to remove cells and suspended particles. The cell-free filtrate was acidified with 1 mL of 1 M hydrochloric acid and frozen at -20 C until analysis. Sampling collection and processing in both May and August occurred mid-morning before local apparent noon.

Dissolved bacimethrin was extracted from lake water using solid phase extraction and its concentration was determined with liquid chromatography mass spectrometry (LC-MS) using a slight modification of previously described methods (6). Briefly, samples were thawed, pH adjusted to 6.5, and bacimethrin was extracted using C_18_ resin (Agilent Bondesil HF). Bacimethrin was eluted from the C_18_ resin using 12 mL of methanol, and further concentrated by nitrogen drying to a volume of 250 L. A 1:1 chloroform liquid phase extraction was used to remove hydrophobic compounds from the sample matrix. Analysis was conducted using an Applied Biosystems 4000 Q-Trap triple quadrupole mass spectrometer with an ESI interface coupled to a Shimadzu LC-20AD liquid chromatograph. Chromatography and mass spectrometer conditions are described elsewhere (7). Similar to the analyses performed at Cornell, bacimethrin was observed to have column retention time of 2.89 minutes, a parent m/z of 156.1, and daughter product m/z of 138.0, 95.0, and 81.0. The declustering potential used was 20 V and the collision energy was 25 V. Samples were analyzed in triplicate and were randomized prior to analysis. An internal standard (^13^C-labeled thiamin) was used for quantification. A known concentration of the IS was spiked into all samples and analytical standards (external curves). The concentration of bacimethrin in samples was determined using the relationship between the observed peak areas of bacimethrin and the IS in the samples compared to the observed peak areas of these compounds in the external curves where the concentrations are known. The inclusion of the internal standard allowed for concentrations to be corrected for matrix effects. Analysis was conducted at the Oregon State University Mass Spectrometry Center.

1. Kim BJ, Richter LV, Hatter N, Tung C-K, Ahner BA, Wu M. 2015. An array microhabitat system for high throughput studies of microalgal growth under controlled nutrient gradients. Lab on a Chip 15:3687-3694.

2. Nemeria NS, Shome B, DeColli AA, Heflin K, Begley TP, Meyers CF, Jordan F. 2016. Competence of thiamin diphosphate-dependent enzymes with 2′-methoxythiamin diphosphate derived from bacimethrin, a naturally occurring thiamin anti-vitamin. Biochemistry 55:1135-1148.

3. Brenna E, Crotti M, Gatti FG, Marinoni L, Monti D, Quaiato S. 2017. Exploitation of a multienzymatic stereoselective cascade process in the synthesis of 2-methyl-3-substituted tetrahydrofuran precursors.  Journal of Organic Chemistry 82:2114-2122.

4. Zhang Q, Sun P, Zheng G, Wang Y, Wang X, Wei H, Xiang W. 2012. Design and synthesis of 3-(2-ethyl-4-{2-[2-(4-fluorophenyl)-5-methyloxazol-4-yl]ethoxy}phenyl) propanoic acid: a novel triple-acting ppar α-, γ-, and δ- agonist. Chemistry Letters 41:406-408.

5. Lyi SM, Zhou X, Kochian LV, Li L. 2007. Biochemical and molecular characterization of the homocysteine S-methyltransferase from broccoli (*Brassica oleracea* var. *italica*). Phytochemistry 68:1112-1119.

6. Suffridge CP, Shannon KC, Matthews H, Johnson RC, Jeffres C, Mantua N, Ward AE, Holmes E, Kindopp J, Aidoo M, Colwell FS. 2024. Connecting thiamine availability to the microbial community composition in Chinook salmon spawning habitats of the Sacramento River basin. Applied and Environmental Microbiology. 90(1):e01760-23. doi:10.1128/aem.01760-23.

7. Suffridge CP, Bolaños LM, Bergauer K, Worden AZ, Morré J, Behrenfeld MJ, Giovannoni SJ. 2020. Exploring vitamin B1 cycling and its connections to the microbial community in the North Atlantic Ocean. Frontiers in Marine Science. doi:10.3389/fmars.2020.606342606342. doi:10.3389/fmars.2020.606342.
